# Understanding the cryptic introgression and mixed ancestry of Red Junglefowl in India

**DOI:** 10.1371/journal.pone.0204351

**Published:** 2018-10-11

**Authors:** Mukesh Thakur, Merwyn Fernandes, Sambandam Sathyakumar, Sujeet K. Singh, Ramesh Kumar Vijh, Jianlin Han, Dong-Dong Wu, Ya-Ping Zhang

**Affiliations:** 1 Wildlife Institute of India, Chandrabani, Dehradun,Uttarakhand, India; 2 State Key Laboratory of Genetic Resources and Evolution and Yunnan Laboratory of Molecular Biology of Domestic Animals, Kunming Institute of Zoology, Chinese Academy of Sciences, Kunming, Yunnan, P.R. China; 3 ICAR-National Bureau of Animal Genetic Resources (NBAGR), G.T. Road Bye Pass, Near Basant Vihar, Karnal, Haryana, India; 4 CAAS—ILRI Joint Laboratory on Livestock and Forage Genetic Resources, Institute of Animal Science, Chinese Academy of Agricultural Sciences (CAAS), Beijing, P.R. China; 5 International Livestock Research Institute (ILRI), Nairobi, Kenya; Banaras Hindu University, INDIA

## Abstract

Red Junglefowls (RJFs), the wild progenitor of modern day chickens (DCs), are believed to be in genetic endangerment due to introgression of domestic genes through opportunistic matings with domestic or feral chickens. Previous studies from India reported rare hybridization of RJFs in the wild. However, RJF population genetic structure, pattern of gene flow and their admixture with DC populations are poorly understood at the landscape level. We conducted this study with a large sample size, covering the predicted natural distribution range of RJFs in India. We documented strong evidence of directional gene flow from DCs to free-ranging wild RJFs, with the Northeastern RJF population exhibiting the most genetic variants in their nuclear and mitochondrial genomes, indicating it to be the ancestral population from which early radiation may have occurred. The results provide evidence that landscape features do not act as a barrier to gene flow and the distribution pattern could not be explored due to physical sharing or exchange of wild birds in the past when forests were continuous across RJF range in India.

## Introduction

The polyphyletic origins of Domestic Chickens (DCs, *Gallus gallus domesticus*) is a reason to speculate that gene flow between RJFs (*Gallus gallus murghi*) and DCs is widespread and more frequent than supposed by previous studies [1,2,3] while domestication may have occurred at multiple locations in South and South-East Asia [4,5,6,7]. However, cryptic introgression from domestic or feral DCs to RJFs or viceversa and the transport of DCs amongst different regions obscure the history of these two species. Several studies have suggested physical mixing and gene flow between RJF in the wild and DC populations [4,8,9, 10,11]. Interestingly, Gering *et al*. [12] reported feralisation of *Kauai* chicken through invasive genetics and further raised the issue of *'domestication in reverse'*. In general, hybridization in the absence of reproductive isolation is an inevitable phenomenon and cannot be avoided in cases where domestic and wild congenerics are sympatric [13,14,15,16]. The situation gets complex when hybrid offsprings are reproductively viable and participate subsequent mating across the species. Allendorf *et al*. [17] stated that 5% or less proportion of hybridization in RJFs is an effect of admixture or natural selection whereas another study, based largely on birds reared in captivity and released into the wild, reported rare hybridization between RJFs and DCs in the wild in India [5]. Berthouly *et al*. [6] postulated that their observation of low genetic exchange might be due to sampling bias and reported a fair gene flow from RJF to local Vietnamese chicken populations.

RJFs in India are widely distributed across 51 x 10^5^ km^2^ in 21 States [18]. Further, based on our field observations and monitoring on RJFs in the wild, we often encountered RJFs and DCs feeding in the same flocks in the vicinity of forest habitats [19]. We believe that the threat of hybridization to RJFs with DCs has not been addressed appropriately at a landscape level. In addition, the extent of hybridization between wild RJFs and DCs is stressed to be of importance in the International Union for Conservation of Nature (IUCN) Action Plan for Pheasants (2000). However, IUCN listing of RJFs as “Least Concern”, the non-listing of RJFs on the Convention of International Trade in Endangered Species of Wild Fauna and Flora (CITES) [20], and the present inclusion of RJFs in the Wildlife (Protection) Act, 1972 of India has no provisions to assess the hybridization threat to this speciesdespite a multi-billion dollar poultry industry has evolved through wild RJF. Recent poultry epidemics, such as the one in Hong Kong in 1998 and the ‘bird flu’ in India and other parts of S.E. Asia, could spell doom to the poultry industry and the only fallback option the poultry farmers would eventually be the ‘wild’ RJF [18].

Further, one of the primary premises in present-day conservation programs is to maximize the conservation of genetic diversity available for potential future use. If hybridization of RJFs with and DCs occurrs and continues, it would produce populations which may not be valued for future breeding and conservation purposes under the IUCN guidelines. Considering the importance of conservation concern to safeguard the wild ancestor of DCs, we undertook this study to answer two important questions:

1. Whether or not, the threat of hybridization and genetic exchange between RJF and DC in India is significant or rare as documented by earlier studies.
2. If such hybridization occurs or has occurred in India in the past, whether it is localized with specific distribution pattern and how does it affect the current population genetic structure of RJF?

## Materials and methods

### Sample collection and DNA extraction

**Fig 1:**
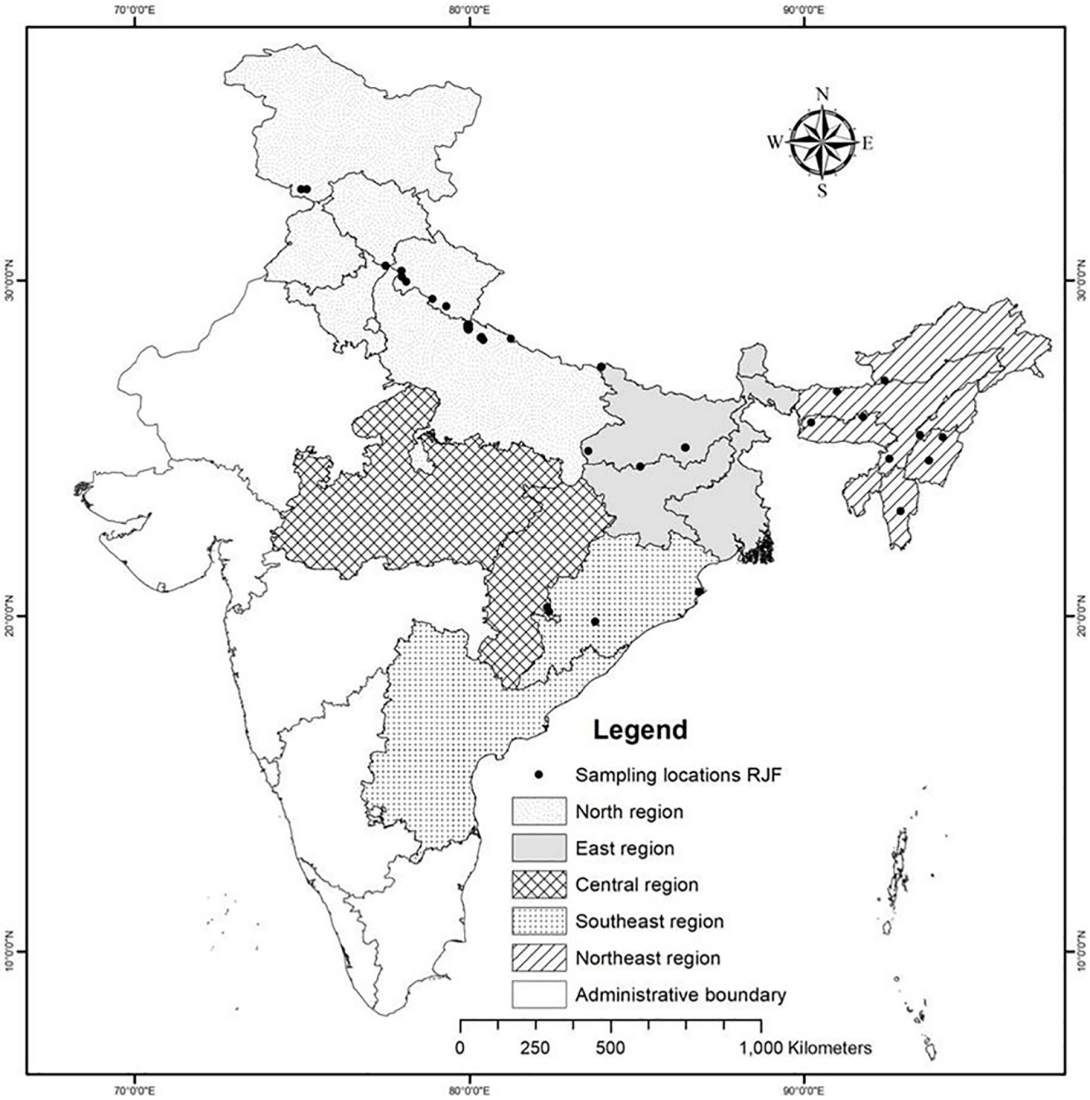
India geographic map showing sampling locations of RJFs.

For sampling, we divided India into five zones based on the availability of continuous habitats for RJF viz., *North* the States of Jammu and Kashmir, Himachal Pradesh, Uttarakhand, Haryana, Punjab and Uttar Pradesh, *Central* the States of Madhya Pradesh and Chhattisgarh, *East* the States of Bihar, Jharkhand, West Bengal and Sikkim, *Southeast* the States of Odisha and Andhra Pradesh and *Northeast* the States of Assam, Arunachal Pradesh, Nagaland, Mizoram, Manipur, Tripura and Meghalaya. We collected blood samples from 57 wild RJF and 79 DC individuals across India (Fig 1). The wild RJF were live trapped and approximately 500μl blood from each bird was withdrawn from the brachial vein and stored in DNA zol BD (Invitrogen TM, Carlsbad, CA, USA). All DC samples were collected in the vicinity of wild RJFs adjacent to the forests. The genomic DNA from whole blood was extracted following Mackey *et al*. [21].

### Microsatellite genotyping and sequencing of D-loop region

We genotyped samples with 30 microsatellite loci (ADL0268, MCW0206, LEI0166, MCW0020, MCW0037, ADL0112, MCW0295, MCW0067, MCW0104, MCW0111, MCW0034, MCW0222, LEI0094, MCW0216, MCW0081, MCW0330, LEI0234, MCW0103, MCW0098, MCW0069, MCW0016, MCW0078, MCW0123, MCW0165, MCW0248, ADL0278, LEI0192, MCW0014, MCW0183 and MCW0284). We scrutinized these loci based on their wide coverage on the genome and high degree of polymorphism [22,23,24]. The PCR composition and cycling conditions followed standard procedures outlined elsewhere [25,26]. The fluorescent-based genotyping was performed through capillary electrophoresis on an ABI 3130 Genetic Analyzer (Applied Biosystems, Foster City, CA, USA). The allele scoring was done manually using GeneMapper (ver. 3.7) (Applied Biosystems, Foster City, CA, USA).

To amplify mitochondrial DNA D-loop hypervariable region (HV1), specific primers L16750 (5′-AGGACTACGGCTTGAAAAGC-3′) as theforward primer and H547 (5′-ATGTGCCTGACCGAGGAACCAG-3′) as reverse primer were used. This primer pair amplified a 550 bp fragment between sites 16750 (GenBank accession number NC_001323; [27]) and 547 (GenBank accession number AB098668; [28]). The PCRs were conducted in 10 μl reaction—0.25 mM MgCl_2_, 1 μM each of forward and reverse primers, 0.25 mM dNTPs and 1 unit *Taq* polymerase. Thermocycling conditions of PCR consisted of an initial denaturation at 94°C for 2 min, followed by 35 cycles of denaturation at 94°C for 30 seconds, an annealing step at 52°C for 20 seconds, and an extension at 72°C for 60 seconds and finished by a final extension at 72°C for 10 min. PCR products were purified using the QIAquick PCR purification kit (QIAGEN, GmbH, Germany) according to the manufacturer’s protocol. Direct sequencing of a HV1 segment of the D-loop region was performed using two internal primers CR-for (5′-TCTATATTCCACATTTCTC-3′) and CR-rev (5′-GCGAGCATAACCAAATGG-3′). Sequencing was done using the BigDye Terminator (ver. 3.1) Cycle Sequencing Kit (Applied Biosystems, Foster City, CA, USA) and the purified sequencing products were electrophoresed on the ABI 3130 Genetic Analyzer.

### Statistical analysis

#### (a) Microsatellites

Genetic variability, inbreeding and population differentiation. Genetic diversity estimates *i*.*e*. observed (Na) andeffective numbers of alleles (ne), observed (Ho), expected (He) and unbiased expected heterozygosity (UHe) and pair‐wise *F*_*ST*_ were estimated using GENEALEX (ver. 6.5) [29]. The mean allelic richness (A_R_), which is an estimate of an independent sample size for each population and the polymorphic information content (PIC)were estimated using FSTAT (ver.2.9.3.2) [30] and Cervus (ver. 3.0) [31], respectively. We tested any deviation from Hardy–Weinberg equilibrium (HWE) at each locus following probability test approach involving 10,000 dememorizations, 500 batches and 10,000 iterations per batch in GENEPOP (ver. 4.2) [32, 33]. The *F*_*IS*_ following Weir and Cockerham [34] method and Linkage disequilibrium (LD) were tested using the a log likelihood ratio statistic in GENEPOP (ver. 4.2) [33].

Population genetic structure. Population genetic structure of RJF in India was inferred using the Bayesian clustering algorithm implemented in program STRUCTURE (ver.2.3.3) [35] following a model assuming *K* populations (e.g. 1 to 10). We selected an admixture model with a burn‐in period of 50,000 and 500,000 Markov chain Monte Carlo repetitions as well as a model of correlated allele frequencies without prior sampling location information. Twenty independent runs at each *K* value were performed. The most appropriate *K* value was determined by calculating ad hoc quantity (ΔK) as proposed by Evanno *et al*. [36] and each individual was assigned to the inferred clusters using a threshold proportion of membership (q), *i*.*e*. q ≥ 0.80, following Mukesh *et al*. [26, 37, 38]. The clustering results of STRUCTURE were visualized over ClumpaK (http://clumpak.tau.ac.il/index.html), a web server that provides a full pipeline for clustering, summarizing and visualizing the STRUCTURE results.

Spatial genetics and barrier detection. As sampling was done across the species distribution in India, we performed a spatial Bayesian clustering analysis in GENELAND (ver.) 4.0.3 [39] to detect barriers in the gene flow between free-ranging RJF populations. Unlike population assignment in STRUCTURE, this analysis also takes into account the spatial coordinates (*i*.*e*. latitude and longitude) of each individual along with their multi-locus genotypes for specifically identifying genetic breaks while generating maps of population ranges. We employed the spatial model to infer the most likely number of clusters indicated as ‘K’ with either correlated or uncorrelated allele frequency model. In these analyses, we conducted 30 independent runs for each K ranging from 1 to 10, with 1,000,000 iterations and 1,000 thinning. Other parameters were set to default values (maximum rate of the Poisson process, 100; uncertainty on spatial coordinates, 0; maximum number of nuclei, 300; null allele model, FALSE). The top three runs showing the highest average logarithm of the posterior probability were selected and post-processing was conducted using 100 x 100 pixels in the spatial domain with a burnin period of 200.

#### (b) Mitochondrial D-loop region

Genetic variability and demographic history. We checked all raw sequences using Sequencher ver. 4.7 (www.genecode.com) and any ambiguity, if encountered, was manually corrected. The cleaned sequences were aligned using CLUSTAL W-multiple sequence alignment algorithm, implemented in BioEdit version 7.2.5 (http://www.mbio.ncsu.edu/BioEdit/bioedit.html). Using DnaSP (ver.5.10) [40], diversity estimates *i*.*e*. polymorphic sites (S), number of haplotypes (H), nucleotide diversity (π), haplotype diversity (Hd), average number of nucleotide differences (K) and mismatch distribution test for demographic expansion, equilibrium or bottleneck [41] were computed. Neutrality tests, *i*.*e*. Tajima’s D [42], Fu’s Fs [43] and Fu and Li’s F and D [44] were carried out to evaluate the demographic effects.

#### (c) Genetic introgression and clustering of RJFs with DCs

Program STRUCTURE with the same parameters as described above was used with the inclusion of DC samples collected in the vicinity of RJFs to assign the admixed individuals. To evaluate the direction of gene flow, haplotypes shared, and their frequencies of occurrence between RJFs and DCs were examined using the phylogenetic network based on the median-joining method [45] implemented inNETWORK ver. 4.5.1 (http://www.fluxusengineering.com). In NETWORK, we assigned equal weights to all variable sites and applied default values for the epsilon parameter (epsilon = 0). The microsatellite and sequencing data phylogenetic trees constructed were used to investigate the recent and long-term admixture and the genetic relationship between wild RJF and DC populations. For microsatellite data, a genetic distance matrix was calculated using Program GENALEX (ver. 6.5) [29], and a phylogenetic tree was constructed following the maximum likelihood (ML) method in MEGA program version 7.0 [46]. For sequencing data, maximum likelihood method and the Tamura 3 parameter, the most fit substitution model, were considered to reconstruct the phylogenetic tree using MEGA program version 7.0 program [46].

## Results

Among the 57 RJF and 79 DC samples collected, there was 68 (32 RJFs and 36 DCs) samples from North, 25 (9 RJFs and 16 DCs) from East, 5 (2 RJFs and 3 DCs) from Central, 5 (4 RJFs and 1 DC) from South East and 33 (10 RJFs and 23 DCs) from Northeast of India. Since, we can trap only a few wild RJFs in Central and Southeast zones; we pooled samples of these zones to create a Cent-Southeast population for further analysis. During analysis, two birds- RJ1 (Cent-Southeast) and RJ9 (Northeast) were excluded due to uncertainty in the GPS locations of their sample points.

We genotyped all samples three times or repeated the process until we generated consensus genotypes for all samples. However, four loci *i*.*e*. LEI0192, MCW0014, MCW0183 and MCW0284 exhibited a considerable amount of missing values for few a samples even after multiple repetition, so we removed them from further analysis. We manually checked allelic data, and found no indication of any genotyping error (Data available on the Dryad Digital Repository on https://doi.org/10.5061/dryad.rv38cp4).

### Genetic diversity indices and demographic history

All 26 microsatellite loci were polymorphic, yielding a total of 692 alleles across the four RJF populations. The average Na and ne ranged from 4.27±0.34 (Cent-Southeast) to 9.69±0.76 (North) and from 3.11±0.27 (Cent-Southeast) to 5.02±0.35 (North), respectively. The Ho ranged from 0.52 (±0.04 North; ±0.06 Cent-Southeast) to 0.58 (±0.05 East; ± 0.04 Northeast) and did not differ significantly among the four populations. The mean PIC values were greater than 0.5 for all the four RJF populations. The UHeranged from 0.67±0.04 in Cent-Southeast to 0.81±0.02 in Northeast population, and the average A_R_ (rarefaction after two diploid individuals per population) ranged from 2.59±0.13 in Cent-Southeast to 3.04±0.07 in Northeast population. Total count of N_P_ was the highest in North (85 alleles) but the lowest in Cent-Southeast (12 alleles) RJF populations. The *F*_*IS*_ estimates in the four RJF populations were significantly positive (P<0.001), suggesting that all the populations were slightly inbred. The number of loci deviated from HWE (P<0.05) ranged from four in Cent-Southeast population to 22 in North population with the majority of them showing a heterozygote deficit. Nevertheless only two loci (MCW0295 and MCW0123) deviated from HWE across the four populations (P<0.05; S1 Table). Several pairs of loci showed significant LD (P<0.05; S2 Table), but none of them was present across the four populations.

For D-loop data, we analysed 100 samples, *i*.*e*.38 wild RJF (15 North, 7 East, 6 Cent-Southeast and 10 Northeast) and 62 DC collected in the vicinity of the RJF. Wild RJF yielded 37 polymorphic sites which formed 31 haplotypes with an average of 6.48 nucleotide differences. All analyzed samples yielded 68 haplotypes, among which 37 were found in DCs. Most haplotypes were present in one sample each. Haplotypes # 1, 12 and 13 were shared among the individuals of the same population while haplotype 3 was shared between North and Northeast populations (S1 Fig). Novel haplotypes were deposited in the GenBank database (Accession no. MG053424 to MG053491). Northeast population represented the highest diversity estimates with 26 polymorphic sites, 10 haplotypes and 8.71 nucleotide differences. The overall haplotype (Hd) and nucleotide diversity (π) were 0.98 ±0.014 and 0.01±0.001, respectively (Table 1). All four RJF populations showed a multimodal pattern of mismatch distribution, supporting these populations to be under demographic equilibrium and without any bottleneck (S2 Fig). The estimates of neutrality tests, in general, showed a similar pattern revealed by mismatch distribution curve while negative estimates of Tajima’s D and Fu’s Fs statistic tests implied that rare alleles were more common than expected. However, the observed estimates were not significant (P>0.10), suggesting that populations have not undergone any expansion. Fu and Li’s D and F tests also indicated no significant departure from neutrality (P>0.10).

**Table 1:** Summary of molecular genetic diversity indices of wild RJF populations.

RJF population (No of msat/mt)†	Diversity estimates (Microsatellites)									Diversity estimates (mt-DNA D-loop)					Neutrality tests			
	Average number of alleles		Average Heterogygosity			A_R_	N_P_	Average PIC	Average F_IS_	S	H	K	Hd	Π	Tajima'sD*	Fu's Fs*	Fu and Li's D*	Fu and Li's F*
	Na	Ne	Ho	He	UHe													
**North** **(32/15)**	9.69 ±0.76	5.02 ±0.35	0.52 ±0.04	0.77 ±0.02	0.79 ±0.02	2.96 ±0.08	85	0.74	0.34	19	11	5.31	0.93 ± 0.05	0.01 ± 0.001	-0.56	-3.07	-0.81	-0.85
**East** **(9/7)**	6.04 ±0.44	4.32 ±0.35	0.58 ±0.05	0.73 ±0.02	0.78 ±0.02	2.93 ±0.09	27	0.69	0.25	17	5	7.00	0.91 ± 0.10	0.02 ± 0.002	-0.26	0.94	-0.27	-0.30
**Cent-Southeast** **(6/6)**	4.27 ±.34	3.11 ±0.27	0.52 ±0.06	0.61 ±0.04	0.67 ±0.04	2.59 ±0.13	12	0.56	0.25	6	6	2.40	1.0 ± 0.09	0.01 ± 0.0008	-0.50	-3.85	-0.42	-0.46
**Northeast** **(10/10)**	6.62 ±0.36	4.74 ±0.30	0.58 ±0.04	0.76 ±0.02	0.81 ±0.02	3.04 ±0.07	22	0.73	0.29	26	10	8.71	1.0 ± 0.04	0.02 ± 0.002	-0.25	-3.89	-0.28	-0.31
**Pooled** **(57/38)**	6.65 ±0.31	4.30 ±0.17	0.55 ±0.02	0.72 ±0.01	0.76 ±0.01	3.09 ±0.06		0.79	0.23	37	31	6.48	0.98 ± 0.014	0.01 ± 0.001	-1.07	-23.14	-1.25	-1.41

Na,- Observed number of alleles; Ne,- Effective number of alleles; Ho,- Observed heterozygosity; He,-Expected heterozygosity; UHe,- Unbiased expected heterozygosity; A_R,_- Allelic richness; N_P,_- Number of private alleles; PIC,- Polymorphic information content; *F*_*IS*,_- Inbreeding coefficient index; S,- Polymorphic sites; H,- Number of haplotypes; K,- Average number of nucleotide differences; Hd,- Haplotypes diversity; Π,- Nucleotide diversity.

† N,- sample size (msat- for microsatellite analysis and / mt- mitochondrial D loop analysis

* P > 0.10 (not significant).

Pair-wise *F*_*ST*_ values between all four RJF populations were significant (P<0.01; Table 2) for both the microsatellite and D-loop data. The highest differentiation was found between North and Cent-Southeast populations (0.10 for microsatellites and 0.30 for D loop data). However, similar estimates were observed between North and Northeast populations for microsatellites (0.05) and D-loop sequences (0.06).

**Table 2:** Comparison of pair-wise *F*_*ST*_ estimates between four wild RJF and four DC populations.

Population	North	East	Cent-Southeast	Northeast
**North**		0.06	0.30	0.06
**East**	0.05		0.11	0.07
**Cent-Southeast**	0.10	0.06		0.27
**Northeast**	0.05	0.07	0.09	

(Fst values below diagonal- microsatellites and Fst values above diagonal mt-DNA- D loop)

### Inference of population genetic structure and clustering patterns on the spatial scale

**Fig 2:**
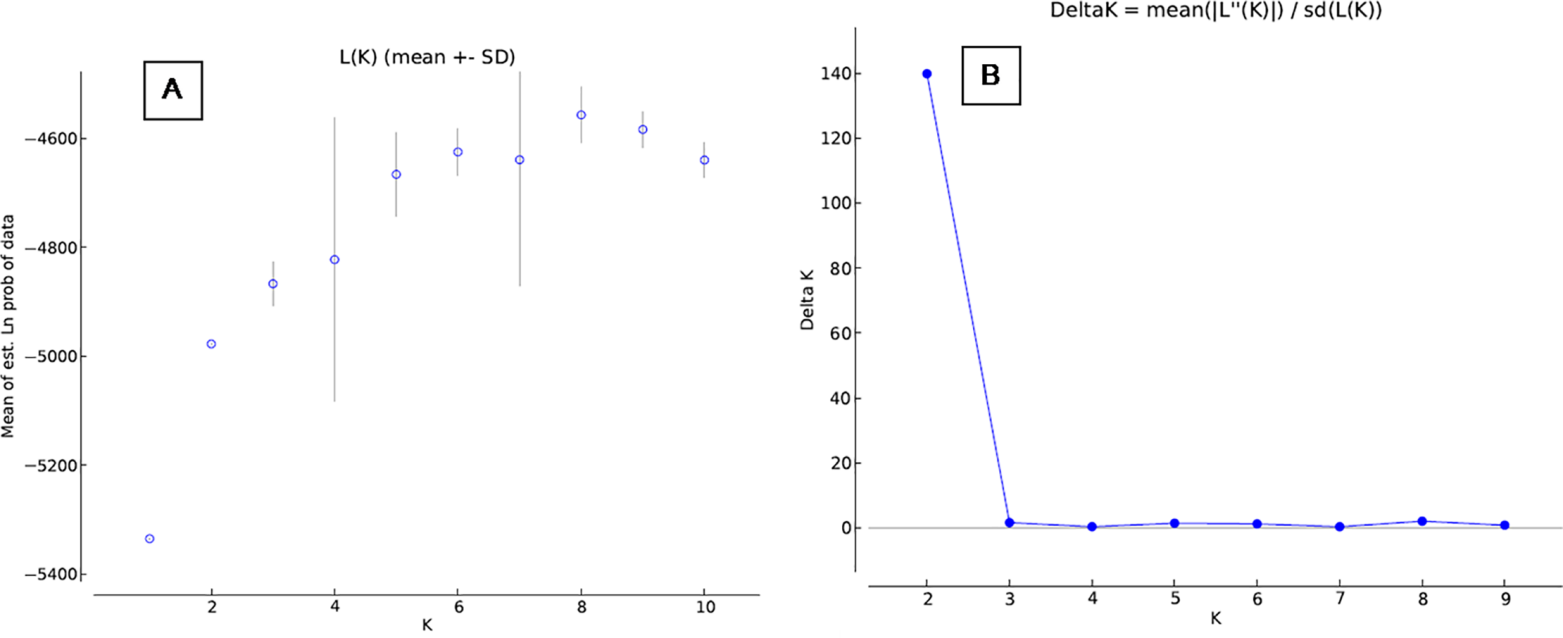
Graphical representation of the true number of the cluster in wild RJF populations. (A) Mean L(K) (±SD) over 20 independent runs for each *K*-value. (B) The ad hoc quantity (ΔK).

**Fig 3:**
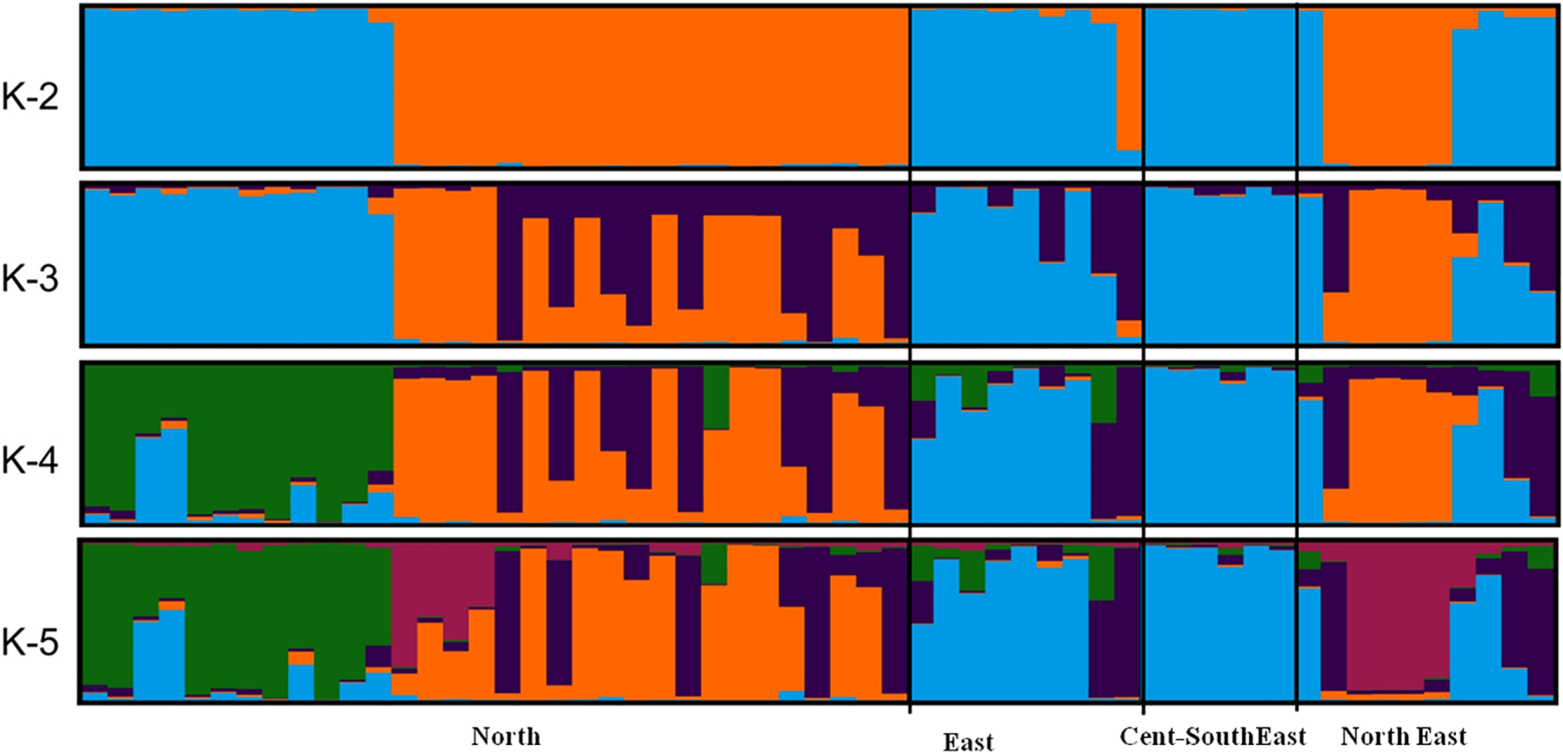
Bayesian clustering patterns of wild RJF populations (n = 57).

The *ad hoc* quantity value was the highest at K = 2 (ΔK -139.8081; Fig 2B; Table 3). All individuals were successfully assigned to two clusters, *cluster 1–*54.38% and *cluster 2–*45.61% based on their posterior probability values *i*.*e*. q ≥ 0.80. A further increase in the *K* value did not split the samples into additional clusters (Fig 3). Interestingly, birds from North and Northeast populations were assigned to both clusters, indicating their shared ancestry in the past. However, nearly all birds of East and Cent-Southeast populations were assigned to *cluster* 1.The global performance of STRUCTURE in assigning all individuals at K = 2 was 100% (Table 3).

**Table 3:** Genetic assignment of wild RJF (n = 57) populations through Bayesian clustering analysis.

Population (No.)	Cluster 1	Cluster 2
North (32)	0.375 (12)	0.625 (20)
East (9)	0.888 (8)	0.111 (1)
Cent-Southeast (6)	1 (6)	0
Northeast (10)	0.5 (5)	0.5 (5)

**Fig 4:**
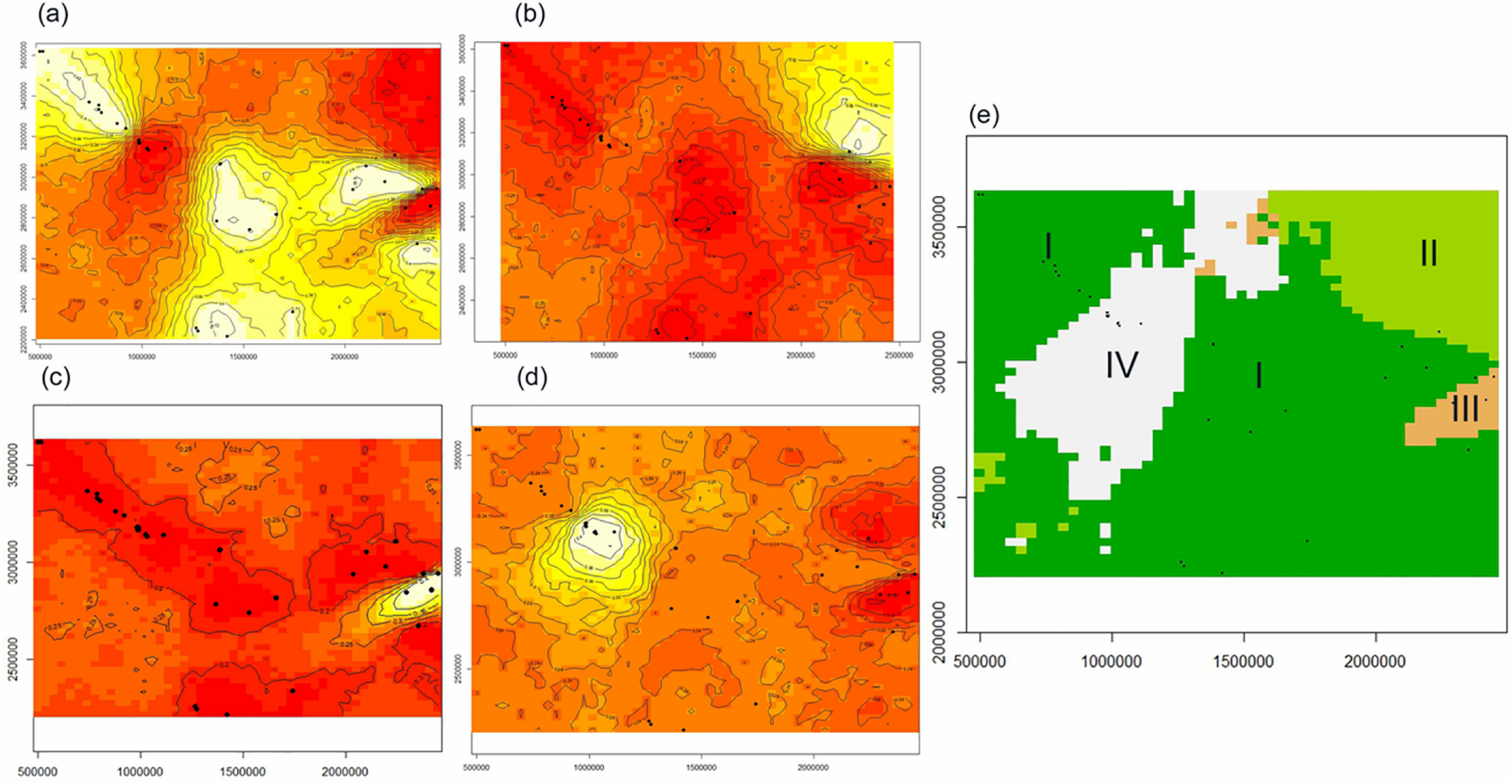
Results of Bayesian model based clustering in GENELAND using wild RJF individuals (n = 57; K = 4 as the best run showing the highest average logarithm of the posterior probability). (a—d) Maps of posterior probability for each inferred cluster show the spatial location of genetic discontinuities of clusters I,II,III and IV; (e) A map of estimated cluster membership shows spatial distribution of the four inferred genetic clusters across the study areas. Sample locations are represented by *black dots*. *X* and *Y spaces* indicate longitude (E) and latitude (N). *Light yellow coloring* corresponds to high posterior probability and *red* corresponds to low posterior probability.

In a GENELAND analysis assuming uncorrelated allele frequency model, four genetic clusters (K = 4) were inferred in 20 out of 30 runs (66.66%; S4 Table, the first three columns,) based on the highest average logarithm of the posterior probability (S3 Fig). In contrast, when assuming correlated allele frequency model, nearly all runs (29/30) indicated 10 clusters (S4 Table, the last three columns) which were not interpretable. Therefore, we considered only the results of the analysis assuming uncorrelated frequency model. The results of the top three runs showing the highest average logarithm of the posterior probability were largely consistent. The spatial distribution of these four inferred clusters showed that the boundaries of these clusters were generally coincided with multiple landscape features and individuals were assigned to these four clusters as cluster-I (green; 36.36%), cluster-II (light green; 7.27%), cluster-III (orange; 5.45%) and cluster- IV (white; 50.90%) (Fig 4E). Interestingly, all the samples collected from the State Uttar Pradesh (Pilibhit Tiger Reserve, Dudhwa National Park, Katerniaghat & Kishanpur Wildlife Sanctuary) were assigned to cluster- IV while samples collected from the State Uttarakhand (Terai Forest Division, Corbett National Park, Rajaji National Park & Wildlife Institute of India campus) were assigned to cluster- I. All the samples collected from Manipur were grouped in cluster-III (S4 Table). Only three individuals (one each from Khelma Forest Division-Nagaland, Hailakandi Forest Division -Assam and Nameri National Park -Assam) were grouped in cluster- II. Samples collected from several locations were assigned to cluster-IV, and this can plausibly be interpreted based on the possible historic gene flow and physical exchange of the birds in the past. Each individual’s estimated proportions of membership obtained from GENELAND analysis (around 0.50 at maximum, S5 Table) were lower than those obtained using the STRUCTURE analysis. The results provided indications that the present landscape features did not act as a barrier to gene flow or the actual geo-spatial clustering trend in the landscape remained masked by the fact of physical sharing/exchange of wild birds in the past as the forested areas and most preferred habitats for the species may be connected.

### Genetic introgression and identification of admixed individuals

**Fig 5:**
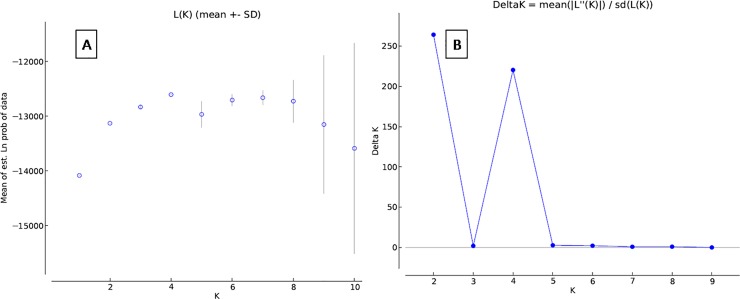
Graphical representation of the true number of the cluster in wild RJF and DC populations. (A) Mean L(K) (±SD) over 20 independent runs for each *K*value. (B) The ad hoc quantity (ΔK).

**Fig 6:**
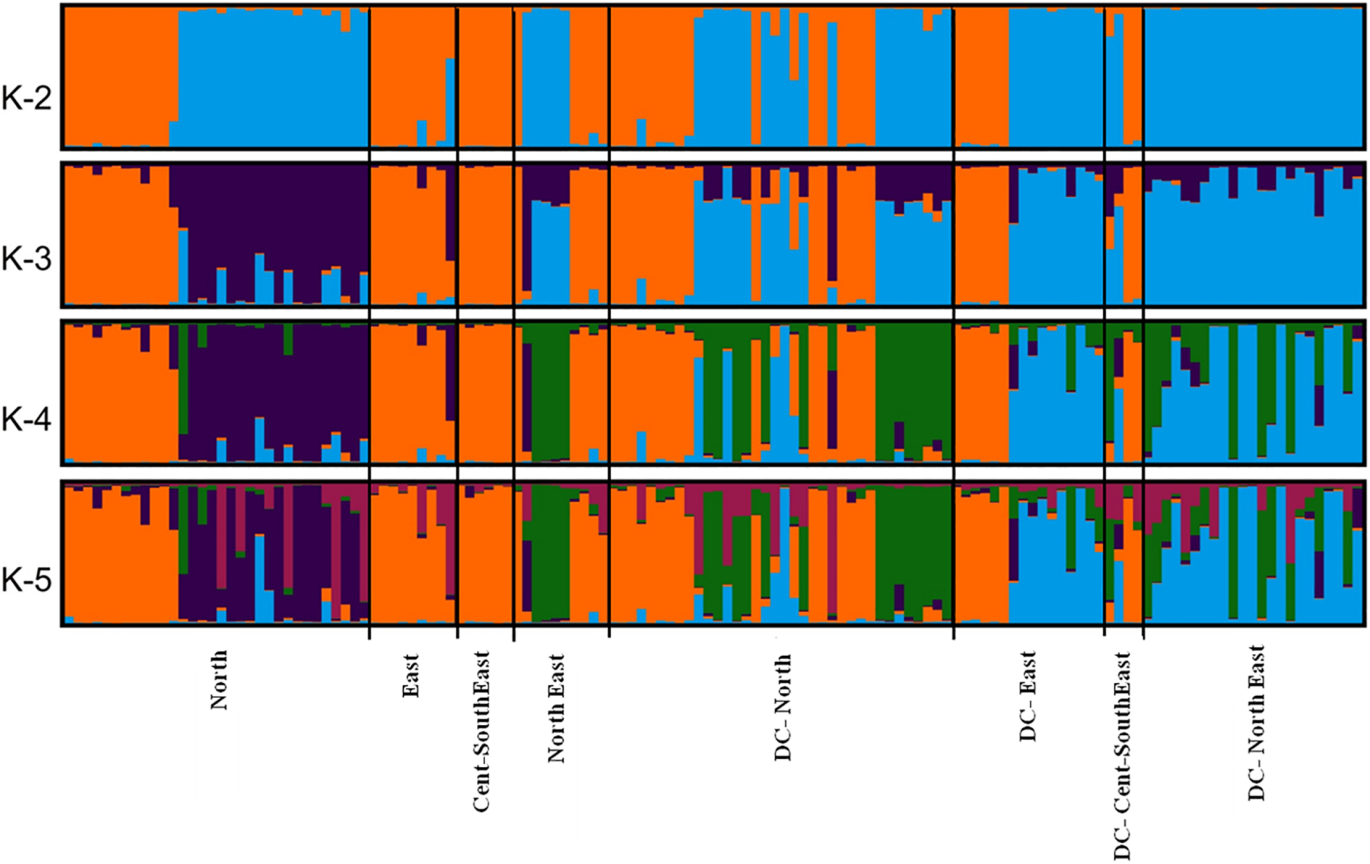
Bayesian clustering patterns of wild RJF (n = 57) and DC(n = 79) populations.

The results indicated a multi-modal value of delta *K* as each peak breaking out quite clearly at *K* = 2 and *K* = 4 (Fig 5B). Individual assignment was >98% at *K* = 2 (Fig 6; S3 Table) and ~82% at *K* = 4 (Fig 6; Table 4) considering a posterior probability value q ≥ 0.80. At *K* = 2, all the RJFs but one bird of East population (RJ-110_SK) were assigned to any of these two clusters while at *K* = 4 nine RJFs were admixed (RJ-76_UK, RJ-84_UK, RJ-116_UP, RJ-55_UP, RJ-58_UP, RJ-63_UP, RJ-105_BH, RJ-110_SK and RJ-113_AS).

**Table 4:** Assignment of admixed individuals between wild RJF (n = 57) and DC (n = 79) populations through Bayesian clustering analysis at *K* = 4.

	Cluster 1	Cluster 2	Cluster 3	Cluster 4	Unassigned individuals	Assigned individuals	Percent assignment
RJF_North (32)	0	16	11	1	4	28	87.50
RJF_East (9)	0	0	7	0	2	7	77.78
RJF_Central_Southeast (6)	0	0	6	0	0	6	100.00
RJF_Northeast (10)	0	0	5	4	1	9	90.00
DC_North (36)	2	0	15	13	6	30	83.33
DC_East (16)	7	0	6		3	13	81.25
DC_Central_Southeast (4)	0	0	2	1	1	3	75.00
DC_Northeast (23)	11	0	0	4	8	15	65.22
Global assignment						111	81.62

**Fig 7:**
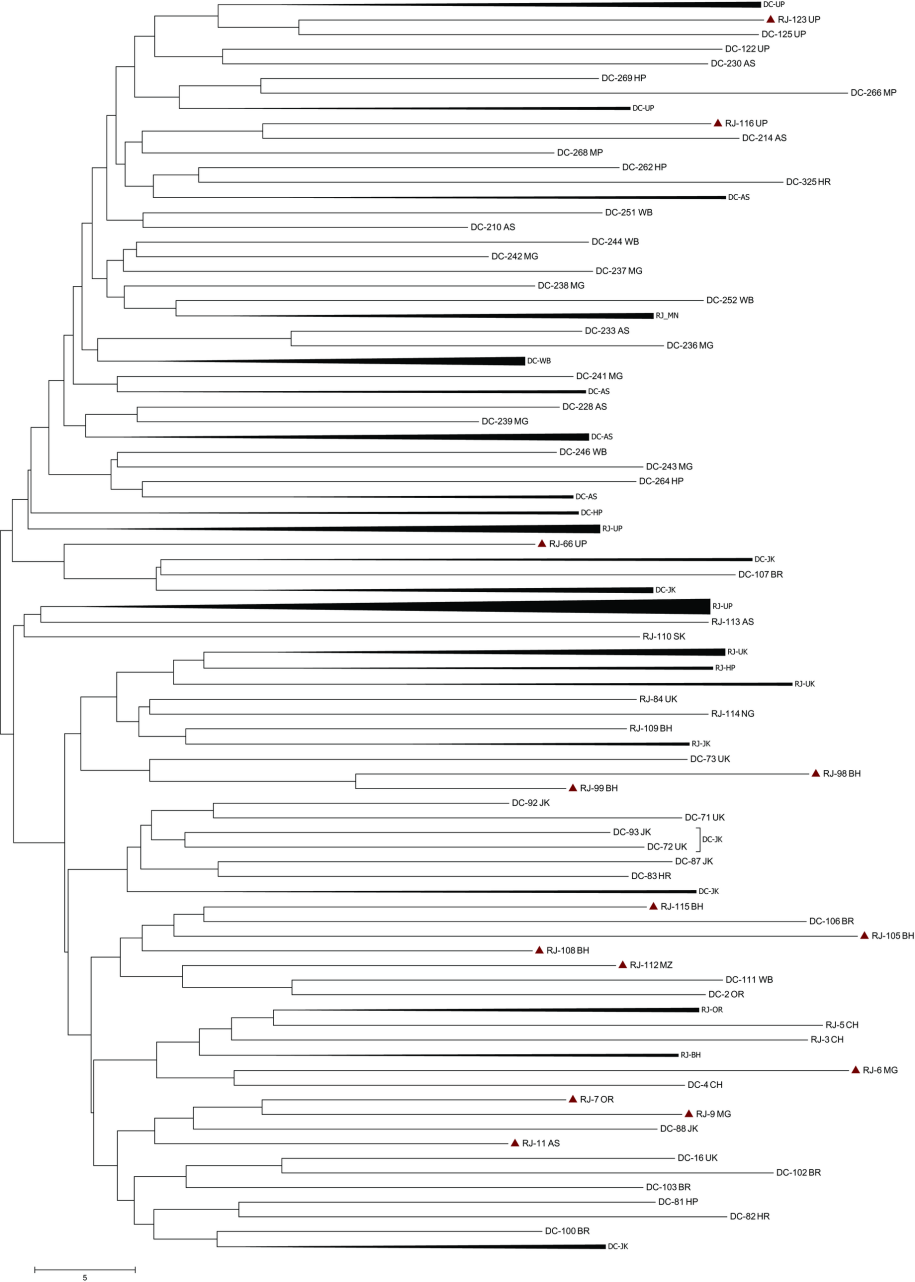
A maximum likelihood phylogenetic tree constructed using microsatellite data of wild RJF and DC samples. (The genetic distance matrix was calculated using GeneAlex and phylogenetic tree was constructed in Mega version 7.0. Scale indicates the allele frequency. The possible hybrids as revealed by sharing similar alleles are represented with red triangle under pink shadow).

**Fig 8:**
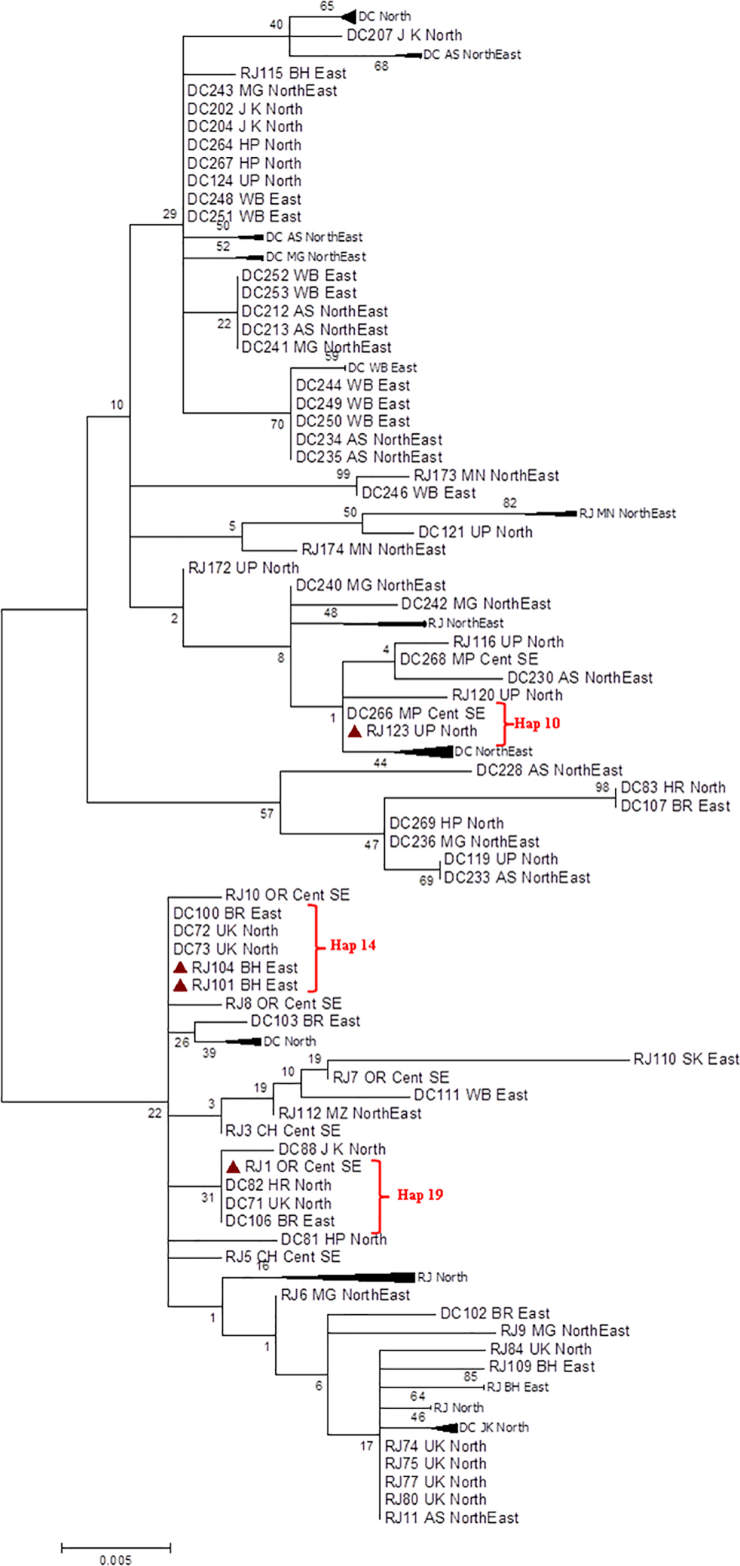
Molecular phylogenetic analysis by Maximum Likelihood method based on the Hasegawa-Kishino-Yano model (HKY+G+I to be the best fit model). (The tree is drawn to scale, with branch lengths measured in the number of substitutions per site. The analysis involved 108 nucleotide sequences. All positions containing gaps and missing data were eliminated. There were a total of 452 positions in the final dataset. The possible hybrids as revealed by sharing the same haplotypes *i*.*e*. Hap# 10, 14 and 19 are represented with red triangle, supporting a longer term admixture).

A Maximum Likelihood (ML) tree obtained from the microsatellite data formed several groups of possible hybrids between the RJFs and DCs (represented by a red triangle with pink shadow in Fig 7. There were 13 RJFs (22.8% of total RJF samples; RJ-123, RJ-116, RJ-66, RJ-98, RJ-99, RJ-115, RJ-105, RJ-108, RJ-112, RJ-6 and RJ-70, RJ-9 and RJ-11) being grouped with DCs representing sharing of similar alleles between RJFs and DCs. This clustering pattern as revealed by ML tree based on a distance matrix of microsatellite data exhibited the recent event of admixture. While ML tree based on D-loop sequences identified several RJFs *i*.*e*. RJ123, RJ101, RJ104 and RJ1 sharing identical haplotypes present in DCs and several other RJFs being grouped with DCs (Fig 8).

**Fig 9:**
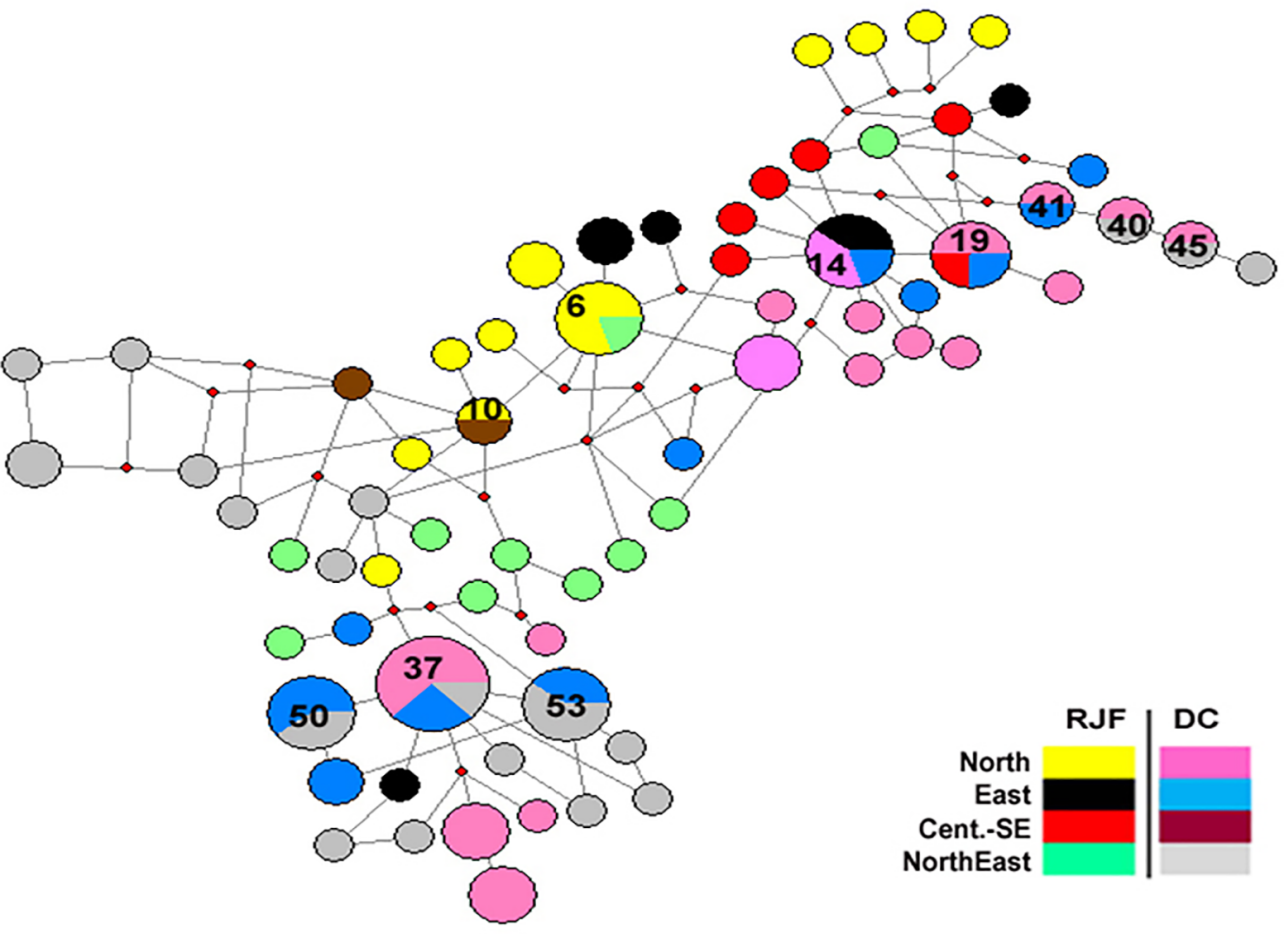
Median joining haplotype network of 136 birds (57 wild RJFs and 79 DCs) belonged to 68 haplotypes. (Haplotypes of RJFs and DCs are represented in different colored circles. The size of the circle is proportional to the haplotype frequency. Important haplotyoes shared within wild RJF populations, between wild RJFs and DCs and within DC populations are numbered).

The median-joining network analysis of 68 haplotypes suggested a strong geospatial structure in the wild RJFs and DCs (Fig 9). To understand the direction of gene flow, we prioritize shared haplotypes over the haplotypes unique to individual populations. We assigned shared haplotypes into three categories *viz*. shared—between RJF and DC populations (Hap#10, 14 and 19), between RJF populations (Hap#6) and between DC populations (Hap#37, 40, 41, 45, 50 and 53). Among three haplotypes shared between RJFs and DCs, Hap#14 and 19 were predominant in DCs at 60% and75%, respectively. While Hap#10 was shared by 50% in RJFs or DCs. In the second group, Hap#6 which was shared between RJF populations of North (Uttarakhand) and Northeast (Assam) might be the ancestral haplotype reflecting historic gene flow between these two populations. Interestingly, it was well supported by the results of STRUCTURE (50% of assignment of Northeast RJFs with North RJFs; Table 3) and GENELAND (One RJF sample collected from Assam being grouped in cluster-IV along with RJFs collected from Uttarakhand; S5 Table) analyses. However, we did not give much attention to the haplotypes shared between DCs as this could be due to the transport of DCs across the country for the local benefit or commercial purposes.

## Discussion

All the 26 loci were polymorphic and informative for population genetic analysis as the majority of loci showed a highPIC value > 0.5. Several loci were deviated individually from HWE in different populations e.g. four loci in Cent-Southeast and 22 loci in North RJF populations due to heterozygote deficit following possible inbreeding at regional level. Two loci were deviated from HWE across all four RJF populations attributed to the significant heterozygote deficit. None of the locus pairs showed significant LD, and independent assortment across the four RJF populations. The diversity indices, *e*.*g*. Na, ne, He, average PIC values and N_P,_ were found to be the highest in the North RJF population. However, most of these genetic attributes in a population are likely influenced by the sample size. UHe and A_R_ which are independent of sample size, were were the highest in Northeast population which was also corroborated by the D-loop data. Statham *et al*. [47] reported that the parental population of a widespread free-ranging animal species, like Russian silver fox (*Vulpes vulpes*), tended to represent most of its genetic diversity across the entire range. Thus our study warrants that Northeast RJF population would be the wild progenitor due to its abundance of genetic variants in both nuclear and mitochondrial genomes from where early radiation of RJFs throughout India would have occurred.

The Bayesian analysis of population assignment grouped all the samples into two clusters and the shared assignment of Northeast RJFs with North RJFs (50% being assigned in *cluster-I*; Table 3) indicated their common ancestry probably due to the habitat connectivity among the two populations along Bhutan, the *Duars* region of North Bengal and the *Terai* region of Nepal and India. However, the GENELAND clustering pattern showed four clusters c (Fig 4E). Interestingly, samples of North RJF population were assigned to both clusters-I and IV (S5 Table). This result was also observed in the STRUCTURE analysis. Further, cluster-II (Manipur) was largely separated from cluster-IV, because of the urban areas while, cluster-III present in a small island, might have been separated from the main range by the Brahmaputra River, a significant zoogeographic barrier as evident for the restricted movement of many terrestrial and arboreal mammals. Only two samples, one each from Sikkim and Assam, were assigned into cluster-III. Cluster-IV revealed interesting observations as individuals were collected from several locations, *i*.*e*. S1 (J&K, HP and UK), S2 (Bihar), S3 (Chhattisgarh), S4 (Odisha), S5 (Assam) and S6 (Mizoram and Nagaland), reflecting this cluster-IV to be a genetic reservoir and perhaps a source of radiation either due to historic gene flow or physical exchange of the birds.

Interestingly, RJF samples from the State of Uttar Pradesh (Pilibhit Tiger Reserve, Dudhwa National Park, Katerniaghat and Kishanpur Wildlife Sanctuary) were assigned to cluster-IV and RJF samples from the State of Uttarakhand (Terai Forest Division, Corbett National Park, Rajaji National Park and Wildlife Institute of India campus) were assigned to cluster-I, irrespective of their physical proximity. There may be several explanations for this unusual observation, one possiblity might be the restricted exchange of wild RJFs within the region by the locals for upgrading their DCs or the other possible reason could be the anthropogenic activities in this region, particularly in and around Terai East and West along the Gola river which might have affected the movement of free-ranging RJFs within the region, as also demonstrated for other animal species such as tigers and leopards [48]. After identifying the admixed individuals between RJFs and DCs, we obtained individual assignment >98% at K = 2 (Fig 5B; S3 Table) but we considered assignment above 82% at *K =* .4 yielding a relatively high resolution of individual ancestry for identifying admixed individuals (Fig 5B; Table 4). At K = 4, nine RJFs *i*.*e*. RJ-76, RJ-84, RJ-116, RJ-55, RJ-58, RJ-63, RJ-105, RJ-110 and RJ-113 were found to be admixed. Since microsatellites have high mutation rates, they provide information about recent evolutionary history as compared to the slow mutating genes, for example the mitochondrial genes that provide data about ancient history [45]. To reconstruct the recent past, we used both these marker systems to address the issue of genetic swapping between wild RJFs and DCs. A ML tree based on microsatellites distance matrix identified 13 RJFs (~23% of the sampled RJFs) being grouped with DCs as plausible hybrids. However, the phylogenetic relationship based on the D loop sequences identified several RJFs *i*.*e*. RJ123, RJ101, RJ104 and RJ1 sharingidentical haplotypes with DCs. These result indicated that hybridization of RJF is not a recent phenomenon but may have persisted since the domestication of junglefowls. By cross-checking the hybrids identified based on microsatellites with the Dloop based phylogeny, we identified RJF *i*.*e*. RJ123 to be a hybrid. This observation was not surprising as the microsatellites and D loop follow different modes of genetic inheritance, where the former are involved in recombinations due to their bi-parental inheritance while D-loop located in mitochondria follows a maternal inheritance. Network analysis has also identified three haplotypes (Hap#10, 14 and 19) to be shared between RJFs and DCs, supporting a longer-term admixture of the species. Among these three haplotypes, frequencies of Hap#14 and 19 were 60% and 75% in DCs suggesting gene flow between RJFs and DCs. This trend, however was supported by a small number of samples probably derived from male RJFs crossing with DC hens. This explanation was highly reasonable since we have encountered the mixing behaviour of RJF males with DC hens even in the presence of DC cocks [19]. Our results are in the accordance with the observations made by Gering *et al*. [12] about the origin of *Kauai* chickens whose behaviour and morphology overlap with those of DCs and RJFs, implying these chickens to be feral *G*. *gallus* descendent from recent invasion(s) of DCs into an ancient RJF reservoir.

To infer on the threat status of the species undergoing hybridization, we believe it is necessary to sample across the species range as the phenomenon may alter with the compromised samples or the sampling locality. Kanginakudru *et al*. [5] concluded that hybridization is a rare phenomenon, but we believe that such underestimated patterns was due to the limited number of samples, mostly collected from captivity. This study is the first attempt to address the phenomenon of hybridization across the distribution of the RJF species, and our results did not find landscape features contributing to the population genetic structure, possibly due to the exchange of wild RJFs in the past when forests were continuous across India.

RJF wild populations across the range are under extreme pressure of poaching for local consumption, habitat loss and genetic hybridization with domestic/feral chickens, it is necessary to monitor the presence/absence and abundance of this species in different parts of the distribution range within as well as outside Protected Area network. Conservation breeding of ‘genetically pure’ RJFs may be initiated with inclusion of genetic parameters and captive stocks are required to be scientifically managed for future use such as research and reintroductions.

## Supporting information

S1 Table Hardy–Weinberg equilibrium (HWE) test. (DOC)

S2 Table Linkage disequilibrium (LD) test. (DOC)

S3 Table Assignment of admixed individuals between wild RJF (n = 57) and DC (n = 79) populations through Bayesian clustering analysis at *K*2. (DOC)

S4 Table The most likely number of clusters (*K*) and average logarithm of the posterior probability for each of the 30 runs in GENELAND analysis assuming either uncorrelated or correlated allele frequency models. (DOC)

S5 Table The individual’s proportion of membership of the wild RJFs (n = 55) in each of the four clusters (*K* = 4) inferred by GENELAND analysis using uncorrelated allele frequency model. (DOC)

S1 Fig Median joining haplotype network of 38 wild RJFs that represented 31 haplotypes. (Haplotypes of four RJF populations are represented in different colored circles. The size of the circle is proportional to the haplotype frequency. Haplotype 3 is shared between North and Northeast populations).
(TIF)

S2 Fig Demographic mismatch distribution curve of four RJF populations. A, North; B, East; C, Central-Southeast and D,Northeast.
(TIF)

S3 Fig The number of cluster (*K*) simulated from the posterior distribution of the best run showing the highest average logarithm of the posterior probability in GENELAND analysis. Analysis was conducted under the spatial model with uncorrelated allele frequencyincorporating latitude/longitude.
(TIF)
