# Supplementary material for: Understanding the cryptic introgression and mixed ancestry of Red Junglefowl in India
Source: PLoS One. 2018 Oct 11;13(10):e0204351. doi: 10.1371/journal.pone.0204351 (PMC6188471; doi:10.1371/journal.pone.0204351)
Supplement: S1 Table — (DOC) [file pone.0204351.s001.doc]

Table S1- Hardy–Weinberg equilibrium (HWE) test

| **Locus** | **RJF- North** | | | | **RJF- East** | | | | **RJF-Cent-SouthEast** | | | | **RJF-NorthEast** | | | |
| --- | --- | --- | --- | --- | --- | --- | --- | --- | --- | --- | --- | --- | --- | --- | --- | --- |
|  | **P-val** | **S.E.** | **Fis estimates** | | **P-val** | **S.E.** | **Fis estimates** | | **P-val** | **S.E.** | **Fis estimates** | | **P-val** | **S.E.** | **Fis estimates** | |
|  |  |  | **W&C** | **R&H** |  |  | **W&C** | **R&H** |  |  | **W&C** | **R&H** |  |  | **W&C** | **R&H** |
| ADL0268 | 0.00 | 0.00 | 0.53 | 0.30 | 0.06 | 0.00 | 0.46 | 0.52 | 1.00 | 0.00 | -0.32 | -0.16 | 0.92 | 0.00 | -0.05 | -0.06 |
| MCW0206 | 0.01 | 0.00 | 0.19 | 0.16 | 0.00 | 0.00 | 0.52 | 0.44 | 0.94 | 0.00 | -0.22 | -0.18 | 0.14 | 0.00 | 0.24 | 0.13 |
| LEI0166 | 0.00 | 0.00 | 0.41 | 0.10 | 0.01 | 0.00 | 0.56 | 0.39 | 0.09 | 0.00 | 1.00 | 1.20 | 0.12 | 0.00 | 0.16 | 0.06 |
| MCW0020 | 0.08 | 0.00 | 0.23 | 0.30 | 0.94 | 0.00 | -0.11 | -0.10 | 1.00 | 0.00 | -1.00 | -1.00 | 0.00 | 0.00 | 0.54 | 0.53 |
| MCW0037 | 0.00 | 0.00 | 0.24 | 0.14 | 0.01 | 0.00 | 0.25 | 0.56 | 0.25 | 0.00 | 0.33 | 0.19 | 0.54 | 0.00 | 0.03 | -0.02 |
| ADL0112 | 0.00 | 0.00 | -0.13 | 0.00 | 0.05 | 0.00 | 0.32 | 0.14 | 0.27 | 0.00 | 0.29 | 0.06 | 0.74 | 0.00 | -0.10 | -0.07 |
| MCW0295 | 0.00 | 0.00 | 0.74 | 0.66 | 0.12 | 0.00 | 0.33 | 0.14 | 0.03 | 0.00 | 0.75 | 0.94 | 0.04 | 0.00 | 0.15 | 0.16 |
| MCW0067 | 0.00 | 0.00 | 0.53 | 0.47 | 0.08 | 0.00 | 0.18 | 0.17 | 0.33 | 0.00 | 0.29 | 0.28 | 0.39 | 0.00 | 0.18 | 0.16 |
| MCW0104 | 0.00 | 0.00 | 0.31 | 0.24 | 0.09 | 0.00 | 0.23 | 0.17 | 0.29 | 0.00 | 0.33 | 0.31 | 0.25 | 0.00 | 0.28 | 0.25 |
| MCW0111 | 0.00 | 0.00 | 0.13 | 0.18 | 0.22 | 0.00 | 0.13 | 0.14 | 0.77 | 0.00 | 0.06 | 0.10 | 0.54 | 0.00 | 0.12 | 0.10 |
| MCW0034 | 0.00 | 0.00 | 0.53 | 0.24 | 0.00 | 0.00 | 0.54 | 0.42 | 0.51 | 0.00 | 0.11 | 0.05 | 0.02 | 0.00 | 0.28 | 0.22 |
| MCW0222 | 0.00 | 0.00 | 0.63 | 0.45 | 0.43 | 0.00 | 0.20 | 0.05 | 0.11 | 0.00 | 0.64 | 0.61 | 0.00 | 0.00 | 0.83 | 0.63 |
| LEI0094 | 0.06 | 0.00 | 0.16 | 0.04 | 0.44 | 0.00 | -0.13 | -0.08 | 0.02 | 0.00 | 0.42 | 0.25 | 0.37 | 0.00 | 0.13 | 0.10 |
| MCW0216 | 0.01 | 0.00 | 0.34 | 0.19 | 0.40 | 0.00 | 0.00 | -0.03 | 0.51 | 0.00 | 0.09 | -0.04 | 0.00 | 0.00 | 0.69 | 0.54 |
| MCW0081 | 0.01 | 0.00 | 0.22 | 0.10 | 0.05 | 0.00 | 0.36 | 0.15 | 1.00 | 0.00 | -0.09 | -0.06 | 0.01 | 0.00 | 0.40 | 0.50 |
| MCW0330 | 0.00 | 0.00 | 0.31 | 0.19 | 0.26 | 0.00 | 0.12 | 0.12 | 0.20 | 0.00 | 0.41 | 0.33 | 0.66 | 0.00 | -0.06 | -0.05 |
| LEI0234 | 0.00 | 0.00 | 0.36 | 0.34 | 0.17 | 0.01 | 0.16 | 0.10 | 0.52 | 0.00 | 0.09 | 0.01 | 0.03 | 0.00 | 0.50 | 0.51 |
| MCW0103 | 0.00 | 0.00 | 0.59 | 0.44 | 0.04 | 0.00 | 0.59 | 0.77 | ND | ND | ND | ND | 0.00 | 0.00 | 0.86 | 0.81 |
| MCW0098 | 0.00 | 0.00 | 0.45 | 0.20 | 0.12 | 0.00 | -0.56 | -0.28 | ND | ND | ND | ND | 0.53 | 0.00 | 0.27 | 0.21 |
| MCW0069 | 0.14 | 0.00 | 0.07 | 0.02 | 1.00 | 0.00 | -0.30 | -0.20 | 0.16 | 0.00 | 0.31 | 0.24 | 0.81 | 0.00 | 0.05 | 0.03 |
| MCW0016 | 0.00 | 0.00 | 0.42 | 0.28 | 0.00 | 0.00 | 1.00 | 1.20 | 0.01 | 0.00 | 1.00 | 1.20 | 0.15 | 0.00 | 0.33 | 0.27 |
| MCW0078 | 0.59 | 0.00 | -0.06 | 0.10 | 0.03 | 0.00 | 0.45 | 0.35 | 0.08 | 0.00 | 0.43 | 0.36 | 0.20 | 0.00 | 0.23 | 0.18 |
| MCW0123 | 0.00 | 0.00 | 0.21 | 0.19 | 0.03 | 0.00 | 0.36 | 0.21 | 0.02 | 0.00 | 0.42 | 0.25 | 0.00 | 0.00 | 0.62 | 0.50 |
| MCW0165 | 0.00 | 0.00 | 0.40 | 0.42 | 0.47 | 0.00 | 0.15 | 0.25 | 0.65 | 0.00 | 0.21 | 0.14 | 0.08 | 0.00 | 0.24 | 0.25 |
| MCW0248 | 0.00 | 0.00 | 0.60 | 0.37 | 0.01 | 0.00 | 0.56 | 0.63 | 1.00 | 0.00 | 0.17 | 0.14 | 0.16 | 0.00 | 0.03 | -0.01 |
| ADL0278 | 0.00 | 0.00 | 0.54 | 0.55 | 0.24 | 0.00 | 0.09 | 0.24 | 0.69 | 0.00 | 0.25 | 0.19 | 0.00 | 0.00 | 0.59 | 0.61 |
|  |  |  | 0.34 |  |  |  | 0.25 |  |  |  | 0.25 |  |  |  | 0.29 |  |
