# Supplementary material for: Understanding the cryptic introgression and mixed ancestry of Red Junglefowl in India
Source: PLoS One. 2018 Oct 11;13(10):e0204351. doi: 10.1371/journal.pone.0204351 (PMC6188471; doi:10.1371/journal.pone.0204351)
Supplement: S2 Table — (DOC) [file pone.0204351.s002.doc]

**Table S2- Linkage disequilibrium (LD) test**

| **Locus#1** | **Locus#2** | **P-Value** | **S.E.** | **Switches** | **P-Value** | **S.E.** | **Switches** | **P-Value** | **S.E.** | **Switches** | **P-Value** | **S.E.** | **Switches** |
| --- | --- | --- | --- | --- | --- | --- | --- | --- | --- | --- | --- | --- | --- |
|  |  | **North** | | | **East** | | | **Cent-SouthEast** | | | **NorthEast** | | |
| MCW0295 | MCW0081 | 0.00 | 0.00 | 1186 | 1.00 | 0.00 | 5309 | ND | ND |  | 0.01 | 0.00 | 4492 |
| MCW0098 | MCW0123 | 0.00 | 0.00 | 2845 | ND | ND |  | 0.51 | 0.00 | 25368 | 0.15 | 0.01 | 6539 |
| MCW0037 | MCW0248 | 0.00 | 0.00 | 5216 | 0.56 | 0.01 | 8806 | 1.00 | 0.00 | 9433 | 0.16 | 0.01 | 7656 |
| ADL0268 | MCW0067 | 0.00 | 0.00 | 2538 | 0.20 | 0.01 | 8039 | 0.02 | 0.00 | 12858 | 1.00 | 0.00 | 1988 |
| ADL0268 | MCW0248 | 0.00 | 0.00 | 2634 | 0.05 | 0.00 | 7963 | 1.00 | 0.00 | 12731 | 1.00 | 0.00 | 4336 |
| ADL0268 | MCW0295 | 0.00 | 0.00 | 2012 | 0.52 | 0.01 | 9390 | 1.00 | 0.00 | 11331 | 1.00 | 0.00 | 2898 |
| ADL0268 | MCW0081 | 0.00 | 0.00 | 727 | 1.00 | 0.00 | 6494 | ND | ND |  | 1.00 | 0.00 | 3236 |
| MCW0078 | MCW0248 | 0.00 | 0.00 | 2769 | 1.00 | 0.00 | 4451 | ND | ND |  | 1.00 | 0.00 | 3733 |
| MCW0248 | ADL0278 | 0.00 | 0.00 | 3615 | 1.00 | 0.00 | 7264 | ND | ND |  | 1.00 | 0.00 | 3837 |
| MCW0016 | MCW0123 | 0.00 | 0.00 | 726 | ND | ND |  | 0.39 | 0.01 | 11324 | ND | ND |  |
| MCW0111 | MCW0330 | 0.00 | 0.00 | 1224 | 1.00 | 0.00 | 2148 | ND | ND |  | ND | ND |  |
| MCW0034 | MCW0248 | 0.00 | 0.00 | 3111 | ND | ND |  | ND | ND |  | ND | ND |  |
| MCW0037 | MCW0103 | 0.00 | 0.00 | 17005 | 0.71 | 0.01 | 10738 | 0.66 | 0.01 | 16773 | 0.28 | 0.01 | 7075 |
| MCW0103 | MCW0248 | 0.00 | 0.00 | 13646 | 0.27 | 0.01 | 8308 | 1.00 | 0.00 | 19251 | 0.65 | 0.01 | 6534 |
| MCW0034 | MCW0103 | 0.00 | 0.00 | 6435 | ND | ND |  | ND | ND |  | ND | ND |  |
| MCW0111 | MCW0248 | 0.00 | 0.00 | 3463 | 1.00 | 0.00 | 3496 | ND | ND |  | ND | ND |  |
| MCW0069 | MCW0078 | 0.00 | 0.00 | 1562 | 1.00 | 0.00 | 14973 | ND | ND |  | ND | ND |  |
| MCW0037 | MCW0098 | 0.00 | 0.00 | 7187 | 0.10 | 0.00 | 16143 | ND | ND |  | 1.00 | 0.00 | 9625 |
| MCW0037 | MCW0067 | 0.00 | 0.00 | 8835 | 1.00 | 0.00 | 8250 | 1.00 | 0.00 | 9153 | 1.00 | 0.00 | 3765 |
| MCW0067 | MCW0078 | 0.00 | 0.00 | 2210 | 1.00 | 0.00 | 3821 | ND | ND |  | 1.00 | 0.00 | 2200 |
| MCW0078 | ADL0278 | 0.00 | 0.00 | 1595 | 1.00 | 0.00 | 6174 | ND | ND |  | ND | ND |  |
| MCW0037 | MCW0111 | 0.00 | 0.00 | 4051 | 0.28 | 0.01 | 6988 | ND | ND |  | ND | ND |  |
| MCW0103 | ADL0278 | 0.00 | 0.00 | 7403 | 0.43 | 0.01 | 10912 | ND | ND |  | 0.20 | 0.02 | 4576 |
| MCW0222 | MCW0103 | 0.00 | 0.00 | 7682 | 1.00 | 0.00 | 24707 | 0.40 | 0.00 | 20152 | 0.40 | 0.01 | 11568 |
| ADL0268 | MCW0037 | 0.00 | 0.00 | 4005 | 0.73 | 0.01 | 14342 | 1.00 | 0.00 | 9238 | 1.00 | 0.00 | 3915 |
| MCW0067 | LEI0234 | 0.00 | 0.00 | 1558 | ND | ND |  | 1.00 | 0.00 | 9334 | 1.00 | 0.00 | 1996 |
| MCW0295 | MCW0248 | 0.00 | 0.00 | 4508 | 0.41 | 0.01 | 6043 | 1.00 | 0.00 | 11282 | 1.00 | 0.00 | 5075 |
| MCW0037 | MCW0034 | 0.00 | 0.00 | 3293 | ND | ND |  | ND | ND |  | 1.00 | 0.00 | 3853 |
| ADL0112 | MCW0067 | 0.01 | 0.00 | 3447 | 1.00 | 0.00 | 3747 | 0.08 | 0.00 | 14366 | 1.00 | 0.00 | 2380 |
| MCW0103 | MCW0123 | 0.01 | 0.00 | 6314 | 1.00 | 0.00 | 7103 | 1.00 | 0.00 | 16688 | 1.00 | 0.00 | 5285 |
| MCW0034 | MCW0081 | 0.01 | 0.00 | 1214 | ND | ND |  | ND | ND |  | 0.09 | 0.01 | 3700 |
| MCW0081 | MCW0123 | 0.01 | 0.01 | 811 | 1.00 | 0.00 | 4183 | ND | ND |  | 0.25 | 0.02 | 4364 |
| MCW0206 | LEI0094 | 0.01 | 0.00 | 1489 | 1.00 | 0.00 | 5018 | 1.00 | 0.00 | 6171 | ND | ND |  |
| MCW0103 | MCW0016 | 0.01 | 0.00 | 5265 | ND | ND |  | 1.00 | 0.00 | 16703 | ND | ND |  |
| MCW0016 | MCW0078 | 0.01 | 0.01 | 892 | ND | ND |  | ND | ND |  | ND | ND |  |
| MCW0222 | MCW0248 | 0.01 | 0.00 | 4452 | 0.34 | 0.00 | 33303 | 0.60 | 0.01 | 20081 | 1.00 | 0.00 | 12547 |
| LEI0094 | MCW0248 | 0.01 | 0.00 | 2963 | 1.00 | 0.00 | 6433 | 1.00 | 0.00 | 9157 | ND | ND |  |
| MCW0098 | MCW0248 | 0.01 | 0.00 | 5972 | 0.30 | 0.01 | 10122 | ND | ND |  | 0.73 | 0.01 | 10106 |
| MCW0020 | MCW0111 | 0.01 | 0.01 | 2073 | ND | ND |  | ND | ND |  | ND | ND |  |
| MCW0037 | MCW0330 | 0.01 | 0.00 | 3416 | 1.00 | 0.00 | 6952 | ND | ND |  | 1.00 | 0.00 | 3937 |
| MCW0098 | MCW0078 | 0.01 | 0.00 | 4372 | ND | ND |  | ND | ND |  | 0.39 | 0.01 | 10926 |
| MCW0037 | MCW0078 | 0.01 | 0.01 | 4072 | ND | ND |  | ND | ND |  | 1.00 | 0.00 | 4114 |
| MCW0067 | MCW0098 | 0.01 | 0.00 | 6706 | ND | ND |  | ND | ND |  | 0.33 | 0.02 | 6450 |
| MCW0103 | MCW0098 | 0.01 | 0.00 | 13196 | 0.33 | 0.01 | 16415 | 1.00 | 0.00 | 24948 | 1.00 | 0.00 | 10289 |
| MCW0034 | ADL0278 | 0.01 | 0.01 | 2187 | ND | ND |  | ND | ND |  | ND | ND |  |
| MCW0111 | MCW0078 | 0.01 | 0.01 | 1702 | 1.00 | 0.00 | 2779 | ND | ND |  | ND | ND |  |
| MCW0098 | ADL0278 | 0.01 | 0.00 | 4362 | 1.00 | 0.00 | 12991 | ND | ND |  | 1.00 | 0.00 | 12334 |
| MCW0016 | ADL0278 | 0.02 | 0.01 | 1266 | ND | ND |  | ND | ND |  | ND | ND |  |
| MCW0037 | ADL0112 | 0.02 | 0.01 | 4660 | 0.29 | 0.01 | 6957 | 0.40 | 0.01 | 11238 | 1.00 | 0.00 | 4788 |
| MCW0111 | MCW0098 | 0.02 | 0.01 | 3492 | ND | ND |  | ND | ND |  | ND | ND |  |
| ADL0268 | ADL0112 | 0.02 | 0.01 | 1887 | 1.00 | 0.00 | 6083 | 0.07 | 0.00 | 14271 | 0.28 | 0.02 | 2875 |
| MCW0037 | MCW0222 | 0.02 | 0.00 | 8135 | ND | ND |  | 0.29 | 0.01 | 14175 | 1.00 | 0.00 | 11223 |
| MCW0098 | MCW0165 | 0.02 | 0.01 | 2797 | ND | ND |  | ND | ND |  | 0.66 | 0.01 | 7115 |
| MCW0295 | MCW0034 | 0.03 | 0.01 | 2233 | ND | ND |  | ND | ND |  | 0.05 | 0.01 | 3959 |
| ADL0268 | MCW0034 | 0.03 | 0.01 | 1406 | ND | ND |  | ND | ND |  | 1.00 | 0.00 | 2162 |
| ADL0268 | MCW0098 | 0.03 | 0.01 | 3064 | 1.00 | 0.00 | 19527 | ND | ND |  | 1.00 | 0.00 | 7629 |
| LEI0166 | MCW0067 | 0.03 | 0.01 | 3292 | 1.00 | 0.00 | 3879 | 1.00 | 0.00 | 19359 | 0.11 | 0.02 | 3612 |
| MCW0330 | MCW0103 | 0.03 | 0.01 | 6476 | 0.30 | 0.02 | 6442 | ND | ND |  | 1.00 | 0.00 | 5605 |
| MCW0081 | MCW0098 | 0.03 | 0.01 | 2293 | 0.40 | 0.01 | 11302 | ND | ND |  | 0.19 | 0.01 | 8964 |
| MCW0020 | MCW0123 | 0.03 | 0.01 | 1856 | ND | ND |  | ND | ND |  | 1.00 | 0.00 | 3374 |
| MCW0111 | MCW0103 | 0.04 | 0.01 | 8575 | 1.00 | 0.00 | 6606 | ND | ND |  | ND | ND |  |
| MCW0016 | MCW0248 | 0.04 | 0.02 | 2311 | ND | ND |  | 0.73 | 0.01 | 14005 | ND | ND |  |
| MCW0111 | MCW0222 | 0.04 | 0.01 | 2574 | ND | ND |  | ND | ND |  | ND | ND |  |
| ADL0268 | MCW0078 | 0.04 | 0.01 | 1304 | 0.28 | 0.01 | 6445 | ND | ND |  | 1.00 | 0.00 | 2714 |
| MCW0067 | MCW0016 | 0.04 | 0.02 | 1469 | ND | ND |  | 0.74 | 0.01 | 14287 | ND | ND |  |
| MCW0330 | MCW0123 | 0.04 | 0.02 | 950 | 1.00 | 0.00 | 4202 | ND | ND |  | 1.00 | 0.00 | 3343 |
| MCW0111 | MCW0034 | 0.04 | 0.01 | 2449 | ND | ND |  | ND | ND |  | ND | ND |  |
| MCW0103 | MCW0078 | 0.04 | 0.01 | 7471 | 1.00 | 0.00 | 7379 | ND | ND |  | 1.00 | 0.00 | 5172 |
| MCW0104 | LEI0234 | 0.05 | 0.01 | 1134 | ND | ND |  | ND | ND |  | 1.00 | 0.00 | 3301 |
| MCW0034 | MCW0098 | 0.05 | 0.01 | 3644 | ND | ND |  | ND | ND |  | 0.39 | 0.01 | 10867 |
| MCW0067 | MCW0104 | 0.05 | 0.01 | 2275 | 0.04 | 0.01 | 4114 | ND | ND |  | 1.00 | 0.00 | 2739 |
| MCW0222 | MCW0098 | 0.05 | 0.01 | 4907 | 0.33 | 0.00 | 33360 | ND | ND |  | 0.34 | 0.00 | 19491 |
| MCW0111 | MCW0165 | 0.05 | 0.01 | 2192 | 1.00 | 0.00 | 6153 | ND | ND |  | ND | ND |  |
| MCW0034 | MCW0330 | 0.05 | 0.02 | 1200 | ND | ND |  | ND | ND |  | 1.00 | 0.00 | 2185 |
| MCW0295 | MCW0165 | 0.06 | 0.01 | 3356 | 1.00 | 0.00 | 10503 | 1.00 | 0.00 | 11364 | 1.00 | 0.00 | 5063 |
| MCW0037 | LEI0234 | 0.06 | 0.01 | 3912 | ND | ND |  | 1.00 | 0.00 | 6246 | 1.00 | 0.00 | 4611 |
| MCW0067 | MCW0248 | 0.06 | 0.01 | 4927 | 0.14 | 0.01 | 5151 | 1.00 | 0.00 | 12777 | ND | ND |  |
| MCW0222 | ADL0278 | 0.06 | 0.01 | 2834 | 1.00 | 0.00 | 19377 | ND | ND |  | ND | ND |  |
| MCW0111 | ADL0278 | 0.06 | 0.01 | 2277 | 1.00 | 0.00 | 5071 | ND | ND |  | ND | ND |  |
| LEI0094 | ADL0278 | 0.07 | 0.02 | 1111 | 1.00 | 0.00 | 9690 | ND | ND |  | ND | ND |  |
| MCW0111 | MCW0123 | 0.07 | 0.02 | 1127 | 0.04 | 0.01 | 4163 | ND | ND |  | ND | ND |  |
| ADL0112 | MCW0330 | 0.08 | 0.02 | 1923 | 1.00 | 0.00 | 2704 | ND | ND |  | 0.00 | 0.00 | 2903 |
| ADL0112 | MCW0165 | 0.08 | 0.02 | 2625 | 1.00 | 0.00 | 8304 | 0.74 | 0.00 | 14159 | 1.00 | 0.00 | 4780 |
| MCW0165 | MCW0248 | 0.09 | 0.01 | 2982 | 1.00 | 0.00 | 14272 | 0.24 | 0.01 | 12927 | 0.20 | 0.01 | 7979 |
| MCW0020 | MCW0034 | 0.09 | 0.02 | 2605 | ND | ND |  | ND | ND |  | 1.00 | 0.00 | 2185 |
| MCW0020 | MCW0078 | 0.09 | 0.02 | 2552 | ND | ND |  | ND | ND |  | 1.00 | 0.00 | 2177 |
| ADL0112 | MCW0103 | 0.09 | 0.01 | 10693 | 1.00 | 0.00 | 6875 | 1.00 | 0.00 | 16830 | 0.76 | 0.01 | 5735 |
| MCW0069 | ADL0278 | 0.09 | 0.02 | 1815 | 0.85 | 0.00 | 20323 | ND | ND |  | 0.06 | 0.01 | 2875 |
| MCW0069 | MCW0248 | 0.09 | 0.02 | 3294 | 0.78 | 0.01 | 16512 | ND | ND |  | 1.00 | 0.00 | 3849 |
| MCW0067 | MCW0216 | 0.09 | 0.01 | 3651 | 0.07 | 0.01 | 3873 | 0.40 | 0.01 | 11299 | 0.28 | 0.02 | 4116 |
| MCW0330 | MCW0248 | 0.09 | 0.02 | 2286 | 1.00 | 0.00 | 3562 | ND | ND |  | 1.00 | 0.00 | 4393 |
| LEI0166 | MCW0016 | 0.10 | 0.03 | 1330 | ND | ND |  | 1.00 | 0.00 | 16346 | ND | ND |  |
| MCW0020 | MCW0098 | 0.10 | 0.01 | 4694 | 0.47 | 0.01 | 9503 | ND | ND |  | 1.00 | 0.00 | 7499 |
| MCW0111 | LEI0234 | 0.10 | 0.02 | 1357 | ND | ND |  | ND | ND |  | ND | ND |  |
| MCW0216 | MCW0016 | 0.10 | 0.02 | 1887 | ND | ND |  | 0.19 | 0.01 | 12136 | ND | ND |  |
| MCW0295 | MCW0067 | 0.11 | 0.01 | 6670 | 0.14 | 0.01 | 5366 | 1.00 | 0.00 | 11501 | 1.00 | 0.00 | 2787 |
| MCW0020 | MCW0081 | 0.12 | 0.03 | 1545 | 1.00 | 0.00 | 6112 | ND | ND |  | 1.00 | 0.00 | 3227 |
| LEI0094 | MCW0098 | 0.12 | 0.02 | 2654 | 0.14 | 0.01 | 11173 | ND | ND |  | ND | ND |  |
| MCW0081 | LEI0234 | 0.13 | 0.03 | 625 | ND | ND |  | ND | ND |  | 1.00 | 0.00 | 3015 |
| MCW0034 | MCW0016 | 0.13 | 0.03 | 1074 | ND | ND |  | ND | ND |  | ND | ND |  |
| MCW0067 | MCW0081 | 0.14 | 0.03 | 1852 | 1.00 | 0.00 | 5212 | ND | ND |  | 1.00 | 0.00 | 2657 |
| MCW0067 | MCW0330 | 0.15 | 0.02 | 2697 | ND | ND |  | ND | ND |  | 1.00 | 0.00 | 2064 |
| MCW0020 | LEI0094 | 0.15 | 0.03 | 1646 | 1.00 | 0.00 | 5663 | ND | ND |  | ND | ND |  |
| MCW0081 | MCW0248 | 0.16 | 0.03 | 2026 | 1.00 | 0.00 | 4788 | ND | ND |  | 0.19 | 0.02 | 4325 |
| MCW0206 | MCW0104 | 0.16 | 0.03 | 1249 | 0.15 | 0.01 | 4877 | ND | ND |  | ND | ND |  |
| MCW0034 | MCW0078 | 0.16 | 0.03 | 1482 | ND | ND |  | ND | ND |  | 1.00 | 0.00 | 2830 |
| LEI0166 | LEI0094 | 0.17 | 0.03 | 1505 | 0.23 | 0.02 | 4665 | 1.00 | 0.00 | 16381 | ND | ND |  |
| MCW0222 | MCW0069 | 0.17 | 0.03 | 2315 | 0.17 | 0.00 | 19314 | ND | ND |  | ND | ND |  |
| MCW0067 | MCW0123 | 0.17 | 0.02 | 3569 | 1.00 | 0.00 | 6373 | 1.00 | 0.00 | 9121 | 1.00 | 0.00 | 2517 |
| MCW0222 | MCW0216 | 0.17 | 0.02 | 3572 | 0.33 | 0.00 | 33180 | 1.00 | 0.00 | 14079 | 0.14 | 0.00 | 30813 |
| MCW0330 | MCW0165 | 0.17 | 0.02 | 2132 | ND | ND |  | ND | ND |  | 1.00 | 0.00 | 3867 |
| LEI0166 | MCW0111 | 0.17 | 0.02 | 2055 | 1.00 | 0.00 | 3630 | ND | ND |  | ND | ND |  |
| ADL0112 | ADL0278 | 0.17 | 0.02 | 3462 | 1.00 | 0.00 | 5800 | ND | ND |  | 1.00 | 0.00 | 3778 |
| MCW0206 | MCW0222 | 0.18 | 0.02 | 3084 | 1.00 | 0.00 | 19668 | 1.00 | 0.00 | 14240 | ND | ND |  |
| MCW0081 | MCW0078 | 0.18 | 0.03 | 954 | 0.23 | 0.02 | 4634 | ND | ND |  | 1.00 | 0.00 | 2760 |
| MCW0020 | MCW0222 | 0.18 | 0.02 | 3636 | ND | ND |  | ND | ND |  | ND | ND |  |
| ADL0112 | MCW0111 | 0.19 | 0.03 | 2174 | 1.00 | 0.00 | 2660 | ND | ND |  | ND | ND |  |
| LEI0166 | MCW0037 | 0.19 | 0.02 | 5199 | 1.00 | 0.00 | 6971 | 1.00 | 0.00 | 16460 | 1.00 | 0.00 | 4875 |
| ADL0268 | MCW0103 | 0.19 | 0.02 | 6775 | 0.43 | 0.01 | 13859 | 1.00 | 0.00 | 19731 | 0.38 | 0.02 | 5575 |
| ADL0268 | MCW0104 | 0.19 | 0.03 | 1330 | 0.37 | 0.01 | 10334 | ND | ND |  | 1.00 | 0.00 | 3578 |
| LEI0166 | MCW0103 | 0.19 | 0.01 | 8186 | 0.81 | 0.01 | 8568 | 1.00 | 0.00 | 16666 | 0.27 | 0.01 | 6506 |
| MCW0020 | MCW0067 | 0.20 | 0.02 | 3662 | ND | ND |  | ND | ND |  | 1.00 | 0.00 | 2103 |
| MCW0098 | MCW0016 | 0.20 | 0.02 | 3907 | ND | ND |  | 0.25 | 0.00 | 25122 | ND | ND |  |
| MCW0037 | MCW0016 | 0.20 | 0.03 | 2411 | ND | ND |  | 1.00 | 0.00 | 11288 | ND | ND |  |
| MCW0037 | ADL0278 | 0.21 | 0.02 | 4287 | 1.00 | 0.00 | 10957 | ND | ND |  | ND | ND |  |
| MCW0020 | ADL0278 | 0.21 | 0.02 | 2808 | 1.00 | 0.00 | 8040 | ND | ND |  | 1.00 | 0.00 | 2889 |
| LEI0166 | MCW0078 | 0.21 | 0.03 | 2252 | 1.00 | 0.00 | 4419 | ND | ND |  | 1.00 | 0.00 | 3902 |
| MCW0206 | MCW0078 | 0.21 | 0.03 | 1041 | 1.00 | 0.00 | 3405 | ND | ND |  | ND | ND |  |
| MCW0081 | MCW0103 | 0.22 | 0.02 | 5589 | 0.61 | 0.01 | 7979 | ND | ND |  | 0.35 | 0.02 | 5574 |
| MCW0034 | MCW0222 | 0.22 | 0.02 | 5313 | ND | ND |  | ND | ND |  | 0.13 | 0.01 | 8669 |
| MCW0216 | MCW0078 | 0.22 | 0.03 | 2918 | 1.00 | 0.00 | 4625 | ND | ND |  | 0.20 | 0.02 | 4688 |
| MCW0067 | MCW0034 | 0.22 | 0.02 | 3464 | ND | ND |  | ND | ND |  | 1.00 | 0.00 | 2098 |
| MCW0206 | MCW0081 | 0.23 | 0.04 | 815 | 1.00 | 0.00 | 4624 | ND | ND |  | ND | ND |  |
| MCW0037 | MCW0295 | 0.23 | 0.02 | 5198 | 1.00 | 0.00 | 8439 | 1.00 | 0.00 | 8315 | 1.00 | 0.00 | 7648 |
| MCW0216 | MCW0098 | 0.24 | 0.02 | 4962 | 0.15 | 0.01 | 11284 | 0.50 | 0.00 | 24921 | 0.18 | 0.01 | 8307 |
| ADL0112 | LEI0234 | 0.24 | 0.03 | 1669 | ND | ND |  | 1.00 | 0.00 | 11487 | 1.00 | 0.00 | 2760 |
| MCW0020 | MCW0103 | 0.24 | 0.01 | 9628 | 1.00 | 0.00 | 12257 | ND | ND |  | 0.36 | 0.02 | 5643 |
| LEI0166 | MCW0330 | 0.24 | 0.03 | 1567 | 1.00 | 0.00 | 3621 | ND | ND |  | 1.00 | 0.00 | 4527 |
| MCW0020 | MCW0248 | 0.24 | 0.02 | 4338 | 0.13 | 0.01 | 5267 | ND | ND |  | 1.00 | 0.00 | 4340 |
| MCW0020 | MCW0330 | 0.24 | 0.03 | 1631 | 1.00 | 0.00 | 4116 | ND | ND |  | 1.00 | 0.00 | 2508 |
| ADL0268 | MCW0111 | 0.25 | 0.04 | 1249 | 1.00 | 0.00 | 5194 | ND | ND |  | ND | ND |  |
| MCW0206 | LEI0234 | 0.25 | 0.04 | 850 | ND | ND |  | 1.00 | 0.00 | 6223 | ND | ND |  |
| MCW0104 | MCW0216 | 0.26 | 0.03 | 2477 | 0.01 | 0.00 | 6908 | ND | ND |  | 1.00 | 0.00 | 5563 |
| MCW0222 | MCW0078 | 0.26 | 0.03 | 2313 | 1.00 | 0.00 | 19367 | ND | ND |  | 1.00 | 0.00 | 16071 |
| MCW0295 | MCW0123 | 0.28 | 0.04 | 1733 | ND | ND |  | 1.00 | 0.00 | 8396 | 0.18 | 0.01 | 4646 |
| MCW0034 | LEI0094 | 0.28 | 0.04 | 1298 | ND | ND |  | ND | ND |  | ND | ND |  |
| MCW0222 | MCW0016 | 0.29 | 0.03 | 2221 | ND | ND |  | 0.70 | 0.00 | 16121 | ND | ND |  |
| MCW0020 | MCW0216 | 0.29 | 0.03 | 3744 | 1.00 | 0.00 | 5824 | ND | ND |  | 1.00 | 0.00 | 4954 |
| ADL0268 | MCW0165 | 0.29 | 0.03 | 1458 | 1.00 | 0.00 | 14153 | 1.00 | 0.00 | 12654 | 1.00 | 0.00 | 3815 |
| MCW0037 | MCW0104 | 0.30 | 0.03 | 3367 | 0.55 | 0.01 | 8792 | ND | ND |  | 1.00 | 0.00 | 4163 |
| MCW0098 | MCW0069 | 0.30 | 0.03 | 3086 | 1.00 | 0.00 | 22364 | ND | ND |  | 1.00 | 0.00 | 11084 |
| MCW0103 | MCW0069 | 0.30 | 0.02 | 7605 | 0.62 | 0.00 | 21386 | ND | ND |  | 0.22 | 0.02 | 4671 |
| MCW0067 | LEI0094 | 0.31 | 0.03 | 2718 | 1.00 | 0.00 | 4177 | 1.00 | 0.00 | 9025 | ND | ND |  |
| MCW0037 | LEI0094 | 0.31 | 0.03 | 2961 | 0.20 | 0.01 | 8181 | 1.00 | 0.00 | 6207 | ND | ND |  |
| MCW0330 | MCW0069 | 0.33 | 0.04 | 1124 | 0.29 | 0.01 | 13043 | ND | ND |  | 1.00 | 0.00 | 2172 |
| MCW0103 | MCW0165 | 0.33 | 0.02 | 7602 | 0.40 | 0.01 | 11288 | 1.00 | 0.00 | 19444 | 0.20 | 0.01 | 7998 |
| LEI0234 | MCW0248 | 0.34 | 0.02 | 3627 | ND | ND |  | 1.00 | 0.00 | 9264 | 1.00 | 0.00 | 4038 |
| MCW0206 | MCW0037 | 0.34 | 0.02 | 4336 | 1.00 | 0.00 | 7121 | 1.00 | 0.00 | 6209 | ND | ND |  |
| MCW0020 | MCW0165 | 0.34 | 0.03 | 2278 | ND | ND |  | ND | ND |  | 1.00 | 0.00 | 3770 |
| ADL0112 | MCW0034 | 0.34 | 0.03 | 2083 | ND | ND |  | ND | ND |  | 1.00 | 0.00 | 2697 |
| LEI0094 | MCW0078 | 0.34 | 0.04 | 1236 | 1.00 | 0.00 | 3818 | ND | ND |  | ND | ND |  |
| LEI0094 | MCW0069 | 0.35 | 0.04 | 1172 | 0.43 | 0.01 | 16396 | ND | ND |  | ND | ND |  |
| ADL0268 | MCW0330 | 0.35 | 0.04 | 1302 | 1.00 | 0.00 | 5113 | ND | ND |  | 1.00 | 0.00 | 2557 |
| MCW0295 | MCW0330 | 0.35 | 0.03 | 2090 | 1.00 | 0.00 | 3734 | ND | ND |  | 0.05 | 0.01 | 2785 |
| ADL0112 | MCW0216 | 0.36 | 0.03 | 3583 | 0.26 | 0.02 | 4483 | 0.80 | 0.01 | 12359 | 0.82 | 0.01 | 4963 |
| MCW0123 | MCW0165 | 0.36 | 0.04 | 1499 | ND | ND |  | 1.00 | 0.00 | 9050 | 1.00 | 0.00 | 7103 |
| MCW0295 | MCW0103 | 0.36 | 0.01 | 9417 | 0.82 | 0.01 | 11808 | 1.00 | 0.00 | 16468 | 1.00 | 0.00 | 7217 |
| ADL0112 | MCW0295 | 0.36 | 0.03 | 3777 | 1.00 | 0.00 | 3621 | 0.80 | 0.01 | 12371 | 0.17 | 0.02 | 3270 |
| MCW0034 | MCW0123 | 0.37 | 0.03 | 2261 | ND | ND |  | ND | ND |  | 0.13 | 0.02 | 3609 |
| ADL0112 | MCW0123 | 0.37 | 0.03 | 1796 | ND | ND |  | 1.00 | 0.00 | 11417 | 1.00 | 0.00 | 3859 |
| ADL0112 | MCW0098 | 0.37 | 0.02 | 5849 | 0.46 | 0.01 | 9668 | 0.50 | 0.00 | 25031 | 0.92 | 0.01 | 7642 |
| MCW0034 | LEI0234 | 0.37 | 0.03 | 1655 | ND | ND |  | ND | ND |  | 1.00 | 0.00 | 2566 |
| MCW0067 | MCW0165 | 0.37 | 0.02 | 4884 | 1.00 | 0.00 | 6164 | 1.00 | 0.00 | 12673 | 0.04 | 0.01 | 3915 |
| MCW0020 | MCW0069 | 0.37 | 0.04 | 1942 | 1.00 | 0.00 | 14452 | ND | ND |  | 1.00 | 0.00 | 2142 |
| LEI0234 | MCW0123 | 0.37 | 0.04 | 1505 | ND | ND |  | 1.00 | 0.00 | 6258 | 1.00 | 0.00 | 3221 |
| MCW0081 | MCW0165 | 0.38 | 0.04 | 1145 | ND | ND |  | ND | ND |  | 0.19 | 0.01 | 6953 |
| MCW0104 | MCW0111 | 0.39 | 0.04 | 1692 | 1.00 | 0.00 | 3912 | ND | ND |  | ND | ND |  |
| ADL0268 | MCW0123 | 0.39 | 0.04 | 1105 | 1.00 | 0.00 | 6308 | 1.00 | 0.00 | 9154 | 1.00 | 0.00 | 3365 |
| LEI0234 | MCW0165 | 0.40 | 0.04 | 1889 | ND | ND |  | 1.00 | 0.00 | 9281 | ND | ND |  |
| ADL0112 | MCW0069 | 0.40 | 0.03 | 3012 | 0.71 | 0.01 | 14252 | ND | ND |  | 1.00 | 0.00 | 2760 |
| ADL0268 | MCW0016 | 0.40 | 0.04 | 632 | ND | ND |  | 0.73 | 0.01 | 14328 | ND | ND |  |
| MCW0081 | MCW0330 | 0.41 | 0.05 | 610 | 1.00 | 0.00 | 3687 | ND | ND |  | 0.13 | 0.02 | 3246 |
| LEI0166 | MCW0020 | 0.41 | 0.03 | 2965 | 1.00 | 0.00 | 5286 | ND | ND |  | 1.00 | 0.00 | 4636 |
| MCW0111 | LEI0094 | 0.42 | 0.04 | 1550 | ND | ND |  | ND | ND |  | ND | ND |  |
| LEI0094 | MCW0016 | 0.42 | 0.05 | 738 | ND | ND |  | 0.40 | 0.01 | 11280 | ND | ND |  |
| MCW0081 | MCW0016 | 0.42 | 0.05 | 489 | ND | ND |  | ND | ND |  | ND | ND |  |
| MCW0330 | MCW0098 | 0.42 | 0.03 | 3118 | 1.00 | 0.00 | 9413 | ND | ND |  | 0.54 | 0.01 | 7507 |
| LEI0234 | MCW0069 | 0.42 | 0.04 | 862 | ND | ND |  | ND | ND |  | 1.00 | 0.00 | 2704 |
| MCW0330 | LEI0234 | 0.43 | 0.04 | 904 | ND | ND |  | ND | ND |  | 1.00 | 0.00 | 2497 |
| MCW0295 | LEI0094 | 0.43 | 0.03 | 1456 | 1.00 | 0.00 | 4634 | 1.00 | 0.00 | 8183 | ND | ND |  |
| LEI0166 | MCW0098 | 0.45 | 0.03 | 3906 | 0.28 | 0.01 | 10044 | ND | ND |  | 0.66 | 0.01 | 7081 |
| MCW0111 | MCW0069 | 0.46 | 0.04 | 1746 | 0.29 | 0.01 | 13147 | ND | ND |  | ND | ND |  |
| MCW0222 | LEI0094 | 0.46 | 0.03 | 3009 | ND | ND |  | ND | ND |  | ND | ND |  |
| MCW0123 | MCW0248 | 0.47 | 0.03 | 3149 | 1.00 | 0.00 | 4164 | 1.00 | 0.00 | 9081 | 0.15 | 0.01 | 5285 |
| MCW0295 | MCW0078 | 0.47 | 0.04 | 2047 | 1.00 | 0.00 | 3641 | ND | ND |  | 1.00 | 0.00 | 3825 |
| ADL0268 | MCW0222 | 0.47 | 0.04 | 1452 | 1.00 | 0.00 | 25139 | 0.61 | 0.00 | 19871 | ND | ND |  |
| MCW0111 | MCW0081 | 0.48 | 0.04 | 1146 | 1.00 | 0.00 | 3606 | ND | ND |  | ND | ND |  |
| MCW0295 | MCW0069 | 0.48 | 0.04 | 2387 | 0.68 | 0.01 | 15279 | ND | ND |  | 1.00 | 0.00 | 3752 |
| MCW0020 | ADL0112 | 0.48 | 0.03 | 2395 | 1.00 | 0.00 | 4242 | ND | ND |  | 0.29 | 0.03 | 2986 |
| MCW0067 | MCW0111 | 0.49 | 0.02 | 3209 | 1.00 | 0.00 | 2995 | ND | ND |  | ND | ND |  |
| MCW0078 | MCW0165 | 0.49 | 0.04 | 1474 | 1.00 | 0.00 | 6260 | ND | ND |  | 1.00 | 0.00 | 4070 |
| MCW0104 | MCW0222 | 0.50 | 0.03 | 2437 | 1.00 | 0.00 | 19218 | ND | ND |  | 1.00 | 0.00 | 9325 |
| ADL0112 | MCW0222 | 0.50 | 0.02 | 6740 | 0.17 | 0.00 | 19623 | 0.41 | 0.00 | 20048 | 0.15 | 0.01 | 8687 |
| LEI0094 | MCW0081 | 0.50 | 0.05 | 656 | 0.09 | 0.01 | 5715 | ND | ND |  | ND | ND |  |
| MCW0295 | LEI0234 | 0.50 | 0.03 | 2487 | ND | ND |  | 1.00 | 0.00 | 8187 | 1.00 | 0.00 | 2646 |
| MCW0067 | MCW0103 | 0.50 | 0.02 | 7903 | 0.25 | 0.01 | 7243 | 1.00 | 0.00 | 19639 | ND | ND |  |
| MCW0020 | MCW0016 | 0.51 | 0.04 | 1767 | ND | ND |  | ND | ND |  | ND | ND |  |
| MCW0216 | MCW0069 | 0.51 | 0.03 | 3303 | 0.19 | 0.01 | 17268 | ND | ND |  | 1.00 | 0.00 | 6554 |
| LEI0166 | MCW0216 | 0.52 | 0.03 | 3281 | 1.00 | 0.00 | 5629 | 1.00 | 0.00 | 16759 | 0.17 | 0.01 | 8966 |
| MCW0104 | MCW0123 | 0.53 | 0.04 | 1453 | 1.00 | 0.00 | 9254 | ND | ND |  | 1.00 | 0.00 | 2921 |
| ADL0268 | LEI0234 | 0.54 | 0.04 | 1071 | ND | ND |  | 1.00 | 0.00 | 9335 | 0.14 | 0.02 | 2496 |
| MCW0034 | MCW0069 | 0.54 | 0.04 | 1628 | ND | ND |  | ND | ND |  | 1.00 | 0.00 | 2183 |
| MCW0037 | MCW0123 | 0.55 | 0.03 | 2818 | 0.19 | 0.01 | 8305 | 1.00 | 0.00 | 6413 | 1.00 | 0.00 | 4895 |
| MCW0295 | MCW0016 | 0.57 | 0.04 | 1362 | ND | ND |  | 0.80 | 0.01 | 12242 | ND | ND |  |
| MCW0078 | MCW0123 | 0.57 | 0.04 | 1192 | 1.00 | 0.00 | 4124 | ND | ND |  | 1.00 | 0.00 | 3735 |
| MCW0295 | MCW0111 | 0.57 | 0.03 | 1901 | ND | ND |  | ND | ND |  | ND | ND |  |
| MCW0216 | MCW0248 | 0.57 | 0.03 | 5034 | 0.03 | 0.00 | 5569 | 1.00 | 0.00 | 11487 | 0.60 | 0.01 | 6552 |
| LEI0166 | MCW0069 | 0.57 | 0.04 | 1718 | 1.00 | 0.00 | 16617 | ND | ND |  | ND | ND |  |
| MCW0165 | ADL0278 | 0.58 | 0.03 | 2739 | 0.14 | 0.01 | 9293 | ND | ND |  | 1.00 | 0.00 | 6124 |
| ADL0268 | MCW0206 | 0.58 | 0.04 | 1272 | 0.27 | 0.01 | 6576 | 0.13 | 0.01 | 9183 | ND | ND |  |
| MCW0069 | MCW0123 | 0.58 | 0.04 | 1775 | 0.42 | 0.00 | 28538 | ND | ND |  | ND | ND |  |
| MCW0104 | MCW0069 | 0.58 | 0.04 | 1105 | 0.49 | 0.01 | 15381 | ND | ND |  | 1.00 | 0.00 | 3835 |
| MCW0295 | MCW0098 | 0.58 | 0.02 | 5043 | 1.00 | 0.00 | 9581 | 1.00 | 0.00 | 25052 | 0.08 | 0.01 | 7121 |
| MCW0067 | MCW0069 | 0.59 | 0.04 | 1908 | 1.00 | 0.00 | 14120 | ND | ND |  | 1.00 | 0.00 | 2083 |
| LEI0094 | MCW0330 | 0.59 | 0.04 | 839 | 1.00 | 0.00 | 3809 | ND | ND |  | ND | ND |  |
| MCW0206 | MCW0216 | 0.60 | 0.03 | 2362 | 0.16 | 0.01 | 4567 | 0.18 | 0.01 | 8300 | ND | ND |  |
| ADL0112 | MCW0248 | 0.60 | 0.02 | 3745 | 0.34 | 0.02 | 4139 | 0.07 | 0.00 | 14041 | 1.00 | 0.00 | 4792 |
| MCW0206 | ADL0112 | 0.60 | 0.03 | 2000 | 1.00 | 0.00 | 3306 | 0.42 | 0.01 | 11163 | ND | ND |  |
| ADL0112 | MCW0104 | 0.60 | 0.04 | 1816 | 1.00 | 0.00 | 4585 | ND | ND |  | 1.00 | 0.00 | 4074 |
| MCW0206 | MCW0069 | 0.61 | 0.04 | 1101 | 1.00 | 0.00 | 15032 | ND | ND |  | ND | ND |  |
| LEI0166 | LEI0234 | 0.61 | 0.03 | 1774 | ND | ND |  | 0.66 | 0.00 | 16655 | 0.27 | 0.02 | 4289 |
| MCW0104 | MCW0248 | 0.61 | 0.03 | 2338 | 0.27 | 0.01 | 6389 | ND | ND |  | 1.00 | 0.00 | 3570 |
| MCW0222 | MCW0165 | 0.62 | 0.02 | 3095 | 1.00 | 0.00 | 19562 | 1.00 | 0.00 | 14253 | 1.00 | 0.00 | 12774 |
| LEI0166 | MCW0248 | 0.62 | 0.02 | 3908 | 1.00 | 0.00 | 5321 | 1.00 | 0.00 | 19256 | 0.27 | 0.01 | 6606 |
| MCW0206 | MCW0111 | 0.63 | 0.03 | 1838 | 1.00 | 0.00 | 2710 | ND | ND |  | ND | ND |  |
| LEI0166 | MCW0222 | 0.65 | 0.03 | 2667 | ND | ND |  | 1.00 | 0.00 | 19971 | 0.24 | 0.01 | 11141 |
| MCW0037 | MCW0069 | 0.65 | 0.02 | 4379 | 0.38 | 0.01 | 14879 | ND | ND |  | ND | ND |  |
| MCW0206 | MCW0020 | 0.65 | 0.03 | 2659 | 0.04 | 0.01 | 4112 | ND | ND |  | ND | ND |  |
| ADL0268 | LEI0094 | 0.65 | 0.04 | 936 | 1.00 | 0.00 | 10336 | 1.00 | 0.00 | 9088 | ND | ND |  |
| MCW0104 | MCW0078 | 0.65 | 0.04 | 1390 | 1.00 | 0.00 | 3851 | ND | ND |  | ND | ND |  |
| MCW0222 | MCW0123 | 0.65 | 0.03 | 2392 | ND | ND |  | ND | ND |  | 0.43 | 0.01 | 10241 |
| MCW0037 | MCW0165 | 0.66 | 0.02 | 5251 | 1.00 | 0.00 | 14221 | 1.00 | 0.00 | 9036 | 0.12 | 0.01 | 9346 |
| LEI0094 | MCW0123 | 0.66 | 0.04 | 978 | ND | ND |  | 1.00 | 0.00 | 6161 | ND | ND |  |
| LEI0094 | MCW0103 | 0.66 | 0.02 | 6022 | 0.12 | 0.01 | 12392 | 0.68 | 0.01 | 17027 | ND | ND |  |
| MCW0216 | MCW0123 | 0.66 | 0.03 | 4013 | 1.00 | 0.00 | 5746 | 0.19 | 0.01 | 8318 | 0.11 | 0.01 | 7005 |
| MCW0020 | MCW0037 | 0.66 | 0.02 | 5116 | ND | ND |  | ND | ND |  | 1.00 | 0.00 | 3918 |
| MCW0206 | MCW0098 | 0.66 | 0.02 | 4151 | 0.50 | 0.01 | 9338 | ND | ND |  | ND | ND |  |
| MCW0104 | MCW0016 | 0.67 | 0.04 | 764 | ND | ND |  | ND | ND |  | ND | ND |  |
| MCW0295 | MCW0104 | 0.67 | 0.03 | 2094 | 0.10 | 0.01 | 5763 | ND | ND |  | 1.00 | 0.00 | 3787 |
| MCW0104 | MCW0330 | 0.68 | 0.04 | 1052 | 1.00 | 0.00 | 3812 | ND | ND |  | 1.00 | 0.00 | 3480 |
| ADL0268 | MCW0020 | 0.68 | 0.03 | 2050 | 1.00 | 0.00 | 9252 | ND | ND |  | 0.09 | 0.02 | 2604 |
| MCW0104 | LEI0094 | 0.68 | 0.04 | 972 | 0.10 | 0.01 | 5722 | ND | ND |  | ND | ND |  |
| MCW0216 | MCW0103 | 0.69 | 0.01 | 14123 | 0.17 | 0.01 | 8875 | 1.00 | 0.00 | 16907 | 0.84 | 0.01 | 7782 |
| MCW0206 | LEI0166 | 0.69 | 0.03 | 1641 | 1.00 | 0.00 | 4272 | 1.00 | 0.00 | 16633 | ND | ND |  |
| MCW0037 | MCW0081 | 0.70 | 0.03 | 2490 | ND | ND |  | ND | ND |  | 0.20 | 0.01 | 6803 |
| MCW0216 | MCW0330 | 0.70 | 0.03 | 2361 | 1.00 | 0.00 | 3598 | ND | ND |  | 0.48 | 0.02 | 4993 |
| MCW0081 | MCW0069 | 0.70 | 0.04 | 851 | 1.00 | 0.00 | 13380 | ND | ND |  | 1.00 | 0.00 | 3633 |
| MCW0069 | MCW0165 | 0.70 | 0.03 | 1949 | 1.00 | 0.00 | 14024 | ND | ND |  | 1.00 | 0.00 | 5719 |
| MCW0330 | MCW0078 | 0.70 | 0.04 | 893 | 1.00 | 0.00 | 2684 | ND | ND |  | 1.00 | 0.00 | 2749 |
| MCW0034 | MCW0165 | 0.72 | 0.03 | 2177 | ND | ND |  | ND | ND |  | 1.00 | 0.00 | 5799 |
| MCW0104 | MCW0098 | 0.73 | 0.02 | 3109 | 0.39 | 0.01 | 11190 | ND | ND |  | 1.00 | 0.00 | 7276 |
| MCW0206 | MCW0067 | 0.74 | 0.03 | 2014 | 0.08 | 0.01 | 3903 | 0.13 | 0.01 | 9489 | ND | ND |  |
| MCW0034 | MCW0216 | 0.74 | 0.02 | 3308 | ND | ND |  | ND | ND |  | 0.29 | 0.02 | 6508 |
| ADL0268 | LEI0166 | 0.74 | 0.03 | 2247 | 1.00 | 0.00 | 7844 | 1.00 | 0.00 | 19202 | 1.00 | 0.00 | 4586 |
| MCW0206 | MCW0248 | 0.74 | 0.02 | 3949 | 0.00 | 0.00 | 4380 | 1.00 | 0.00 | 9329 | ND | ND |  |
| LEI0166 | ADL0278 | 0.76 | 0.03 | 1745 | 0.48 | 0.01 | 7398 | ND | ND |  | ND | ND |  |
| LEI0234 | MCW0098 | 0.76 | 0.02 | 3825 | ND | ND |  | 1.00 | 0.00 | 25205 | 1.00 | 0.00 | 6553 |
| ADL0112 | MCW0081 | 0.77 | 0.03 | 1376 | 0.10 | 0.01 | 3676 | ND | ND |  | 0.38 | 0.02 | 3651 |
| LEI0094 | MCW0165 | 0.80 | 0.02 | 1888 | ND | ND |  | 0.13 | 0.01 | 9322 | ND | ND |  |
| MCW0111 | MCW0216 | 0.80 | 0.02 | 3245 | 1.00 | 0.00 | 3688 | ND | ND |  | ND | ND |  |
| ADL0268 | MCW0069 | 0.82 | 0.03 | 1511 | 1.00 | 0.00 | 17351 | ND | ND |  | 1.00 | 0.00 | 2141 |
| MCW0037 | MCW0216 | 0.82 | 0.01 | 5980 | 0.54 | 0.01 | 8733 | 1.00 | 0.00 | 8330 | 1.00 | 0.00 | 12391 |
| MCW0067 | MCW0222 | 0.82 | 0.01 | 4272 | 1.00 | 0.00 | 19379 | 0.60 | 0.01 | 19976 | 1.00 | 0.00 | 8651 |
| MCW0216 | LEI0234 | 0.82 | 0.02 | 2229 | ND | ND |  | 1.00 | 0.00 | 8316 | 0.56 | 0.02 | 4526 |
| ADL0112 | MCW0016 | 0.83 | 0.03 | 1376 | ND | ND |  | 0.61 | 0.01 | 13634 | ND | ND |  |
| MCW0020 | MCW0295 | 0.83 | 0.02 | 2925 | 1.00 | 0.00 | 4172 | ND | ND |  | 1.00 | 0.00 | 2761 |
| MCW0104 | MCW0034 | 0.83 | 0.03 | 1390 | ND | ND |  | ND | ND |  | ND | ND |  |
| MCW0216 | ADL0278 | 0.84 | 0.02 | 2901 | 1.00 | 0.00 | 7782 | ND | ND |  | 1.00 | 0.00 | 7180 |
| LEI0166 | MCW0295 | 0.85 | 0.02 | 3299 | 1.00 | 0.00 | 4350 | 0.50 | 0.01 | 16627 | 1.00 | 0.00 | 6877 |
| MCW0069 | MCW0016 | 0.85 | 0.03 | 936 | ND | ND |  | ND | ND |  | ND | ND |  |
| LEI0234 | MCW0103 | 0.88 | 0.01 | 5710 | ND | ND |  | 0.68 | 0.00 | 16875 | 0.48 | 0.02 | 5245 |
| MCW0295 | MCW0216 | 0.88 | 0.01 | 4470 | 0.31 | 0.01 | 6483 | 1.00 | 0.00 | 10088 | 0.08 | 0.01 | 7376 |
| ADL0112 | MCW0078 | 0.91 | 0.02 | 1813 | 1.00 | 0.00 | 3236 | ND | ND |  | 1.00 | 0.00 | 3164 |
| LEI0166 | MCW0081 | 0.91 | 0.02 | 1246 | 0.39 | 0.02 | 5997 | ND | ND |  | 1.00 | 0.00 | 6047 |
| LEI0094 | MCW0216 | 0.92 | 0.02 | 2097 | 0.19 | 0.01 | 6904 | 0.20 | 0.01 | 8299 | ND | ND |  |
| MCW0020 | MCW0104 | 0.92 | 0.01 | 2175 | 1.00 | 0.00 | 6500 | ND | ND |  | 1.00 | 0.00 | 3450 |
| LEI0166 | MCW0034 | 0.92 | 0.01 | 2025 | ND | ND |  | ND | ND |  | 1.00 | 0.00 | 3977 |
| LEI0166 | MCW0165 | 0.93 | 0.01 | 3108 | 1.00 | 0.00 | 6197 | 1.00 | 0.00 | 19470 | 0.18 | 0.01 | 7820 |
| MCW0216 | MCW0081 | 0.93 | 0.02 | 1867 | 1.00 | 0.00 | 4720 | ND | ND |  | 0.10 | 0.01 | 5970 |
| MCW0104 | MCW0103 | 0.94 | 0.01 | 6419 | 0.07 | 0.01 | 7097 | ND | ND |  | 1.00 | 0.00 | 7340 |
| ADL0268 | MCW0216 | 0.95 | 0.01 | 3682 | 0.40 | 0.01 | 8521 | 0.41 | 0.01 | 11389 | 1.00 | 0.00 | 5121 |
| ADL0112 | LEI0094 | 0.95 | 0.02 | 1606 | 1.00 | 0.00 | 3812 | 1.00 | 0.00 | 11463 | ND | ND |  |
| MCW0295 | MCW0222 | 0.95 | 0.01 | 3315 | 1.00 | 0.00 | 19378 | 1.00 | 0.00 | 15856 | 0.03 | 0.00 | 11297 |
| LEI0166 | MCW0123 | 0.96 | 0.01 | 2202 | 1.00 | 0.00 | 5654 | 0.67 | 0.00 | 16432 | 1.00 | 0.00 | 4374 |
| LEI0166 | ADL0112 | 0.96 | 0.01 | 2789 | 1.00 | 0.00 | 4024 | 1.00 | 0.00 | 16574 | 1.00 | 0.00 | 5081 |
| MCW0111 | MCW0016 | 0.96 | 0.01 | 1202 | ND | ND |  | ND | ND |  | ND | ND |  |
| LEI0166 | MCW0104 | 1.00 | 0.00 | 1703 | 1.00 | 0.00 | 4934 | ND | ND |  | 1.00 | 0.00 | 5460 |
| MCW0222 | MCW0081 | 1.00 | 0.00 | 1776 | ND | ND |  | ND | ND |  | 0.07 | 0.00 | 15558 |
| MCW0222 | MCW0330 | 1.00 | 0.00 | 1735 | ND | ND |  | ND | ND |  | 0.13 | 0.01 | 8852 |
| MCW0222 | LEI0234 | 1.00 | 0.00 | 1843 | ND | ND |  | ND | ND |  | 0.14 | 0.01 | 8819 |
| MCW0216 | MCW0165 | 1.00 | 0.00 | 3207 | 1.00 | 0.00 | 9146 | 0.39 | 0.01 | 11435 | 0.64 | 0.01 | 7176 |
| MCW0020 | LEI0234 | 1.00 | 0.00 | 1824 | ND | ND |  | ND | ND |  | 1.00 | 0.00 | 2460 |
| MCW0104 | MCW0081 | 1.00 | 0.00 | 714 | 1.00 | 0.00 | 5643 | ND | ND |  | 1.00 | 0.00 | 3535 |
| MCW0104 | MCW0165 | 1.00 | 0.00 | 1627 | 1.00 | 0.00 | 6150 | ND | ND |  | 1.00 | 0.00 | 5809 |
| ADL0268 | ADL0278 | 1.00 | 0.00 | 1649 | 1.00 | 0.00 | 8771 | ND | ND |  | 1.00 | 0.00 | 2885 |
| MCW0295 | ADL0278 | 1.00 | 0.00 | 3767 | 1.00 | 0.00 | 9177 | ND | ND |  | 1.00 | 0.00 | 4078 |
| MCW0104 | ADL0278 | 1.00 | 0.00 | 1501 | 1.00 | 0.00 | 6498 | ND | ND |  | 1.00 | 0.00 | 4140 |
| MCW0081 | ADL0278 | 1.00 | 0.00 | 978 | 1.00 | 0.00 | 5972 | ND | ND |  | 1.00 | 0.00 | 3970 |
| MCW0330 | ADL0278 | 1.00 | 0.00 | 1344 | 1.00 | 0.00 | 5008 | ND | ND |  | 1.00 | 0.00 | 2939 |
| LEI0234 | MCW0078 | 1.00 | 0.00 | 799 | ND | ND |  | ND | ND |  | 1.00 | 0.00 | 2625 |
| LEI0234 | ADL0278 | 1.00 | 0.00 | 1365 | ND | ND |  | ND | ND |  | 1.00 | 0.00 | 3649 |
| MCW0206 | MCW0016 | 1.00 | 0.00 | 772 | ND | ND |  | 0.39 | 0.01 | 11263 | ND | ND |  |
| MCW0016 | MCW0165 | 1.00 | 0.00 | 1285 | ND | ND |  | 0.73 | 0.01 | 14148 | ND | ND |  |
| MCW0206 | MCW0295 | 1.00 | 0.00 | 2897 | 0.23 | 0.01 | 4670 | 1.00 | 0.00 | 8481 | ND | ND |  |
| MCW0206 | MCW0103 | 1.00 | 0.00 | 4732 | 0.54 | 0.01 | 7472 | 1.00 | 0.00 | 16646 | ND | ND |  |
| MCW0206 | MCW0123 | 1.00 | 0.00 | 1697 | 1.00 | 0.00 | 4121 | 1.00 | 0.00 | 6246 | ND | ND |  |
| MCW0206 | MCW0165 | 1.00 | 0.00 | 3033 | 1.00 | 0.00 | 9234 | 1.00 | 0.00 | 9160 | ND | ND |  |
| LEI0094 | LEI0234 | 1.00 | 0.00 | 1247 | ND | ND |  | 1.00 | 0.00 | 6327 | ND | ND |  |
| LEI0234 | MCW0016 | 1.00 | 0.00 | 560 | ND | ND |  | 1.00 | 0.00 | 11338 | ND | ND |  |
| MCW0206 | MCW0330 | 1.00 | 0.00 | 1009 | 1.00 | 0.00 | 2738 | ND | ND |  | ND | ND |  |
| MCW0206 | ADL0278 | 1.00 | 0.00 | 1815 | 1.00 | 0.00 | 6132 | ND | ND |  | ND | ND |  |
| MCW0067 | ADL0278 | 1.00 | 0.00 | 2691 | 1.00 | 0.00 | 5177 | ND | ND |  | ND | ND |  |
| MCW0123 | ADL0278 | 1.00 | 0.00 | 2875 | 1.00 | 0.00 | 8142 | ND | ND |  | ND | ND |  |
| MCW0206 | MCW0034 | 1.00 | 0.00 | 2468 | ND | ND |  | ND | ND |  | ND | ND |  |
| MCW0330 | MCW0016 | 1.00 | 0.00 | 546 | ND | ND |  | ND | ND |  | ND | ND |  |
