# Supplementary material for: Understanding the cryptic introgression and mixed ancestry of Red Junglefowl in India
Source: PLoS One. 2018 Oct 11;13(10):e0204351. doi: 10.1371/journal.pone.0204351 (PMC6188471; doi:10.1371/journal.pone.0204351)
Supplement: S3 Table — (DOC) [file pone.0204351.s003.doc]

**Table S3 - Assignment of admixed individuals between wild RJF (n=57) and DC (n=**79) populations through Bayesian clustering analysis at K-2

| **Pop. (n)** | **Cluster 1** | **Cluster 2** | **Unassigned individuals** | **Assigned individuals** | **Percent assignment** |
| --- | --- | --- | --- | --- | --- |
| RJF_North (32) | 20 | 12 |  | 32 | 100.00 |
| RJF_East (9) | 0 | 8 | 1 | 8 | 88.89 |
| RJF_Cent-SouthEast (6) | 0 | 6 |  | 6 | 100.00 |
| RJF_Northeast (10) | 5 | 5 |  | 10 | 100.00 |
| DC_North (36) | 19 | 16 | 1 | 35 | 97.22 |
| DC_East (16) | 10 | 6 |  | 16 | 100.00 |
| DC_ Cent-SouthEast (4) | 2 | 2 |  | 4 | 100.00 |
| DC_Northeast (23) | 23 | 0 |  | 23 | 100.00 |
| Global assignment |  |  |  | 134 | 98.53 |
