# Supplementary material for: Understanding the cryptic introgression and mixed ancestry of Red Junglefowl in India
Source: PLoS One. 2018 Oct 11;13(10):e0204351. doi: 10.1371/journal.pone.0204351 (PMC6188471; doi:10.1371/journal.pone.0204351)
Supplement: S4 Table — (DOC) [file pone.0204351.s004.doc]

**Table S4 The most likely number of clusters (*K*) and average logarithm of the posterior probability for each of the 30 runs in GENELAND analysis assuming either uncorrelated or correlated allele frequency models**

| **Uncorrelated allele frequency model** | | | **Correlated allele frequency model** | | |
| --- | --- | --- | --- | --- | --- |
| Run | The most likely number of clusters | Average log posterior probability | Run | The most likely number of clusters | Average log posterior probability |
| 1 | 3 | -3053.006493 | 1 | 10 | 17619.63 |
| 2 | 4 | -3858.852811 | 2 | 10 | 15572.68 |
| 3 | 4 | -2645.731891 | 3 | 10 | 19034.32 |
| 4 | 5 | -1777.327725 | 4 | 10 | 16442.49 |
| 5 | 4 | -2366.266287 | 5 | 10 | 14461.65 |
| 6 | 4 | -2588.089665 | 6 | 10 | 19369.5 |
| 7 | 3 | -3368.367013 | 7 | 10 | 13312.37 |
| 8 | 4 | -2333.7872 | 8 | 10 | 13417.57 |
| 9 | 5 | -1821.290528 | 9 | 10 | 20835.96 |
| 10 | 5 | -2202.919025 | 10 | 10 | 18219.4 |
| 11 | 4 | -2297.256865 | 11 | 10 | 21957.58 |
| 12 | 4 | -2513.306781 | 12 | 10 | 16891.18 |
| 13 | 5 | -1837.383018 | 13 | 10 | 18104.05 |
| 14 | 4 | -2640.507882 | 14 | 10 | 17908.29 |
| 15 | 4 | -4475.84488 | 15 | 10 | 14926.47 |
| 16 | 5 | -2238.434652 | 16 | 10 | 16306.22 |
| 17 | 4 | -2945.788638 | 17 | 10 | 16267.19 |
| 18 | 3 | -3221.664648 | 18 | 10 | 15094.05 |
| 19 | 4 | -2378.926571 | 19 | 10 | 12488.09 |
| 20 | 4 | -2805.867106 | 20 | 10 | 12936.34 |
| 21 | 4 | -2395.357864 | 21 | 10 | 21615.95 |
| 22 | 4 | -4287.276855 | 22 | 10 | 15256.34 |
| 23 | 4 | -3297.266365 | 23 | 10 | 17720.46 |
| 24 | 5 | -2797.216864 | 24 | 9 | 16620.1 |
| 25 | 4 | -2897.236866 | 25 | 10 | 15693.2 |
| 26 | 4 | -3297.346864 | 26 | 10 | 17907.24 |
| 27 | 4 | -4297.156765 | 27 | 10 | 16805.32 |
| **28** | **4** | **-2286.386865** | 28 | 10 | 15606.22 |
| 29 | 5 | -3297.456765 | 29 | 10 | 14219.25 |
| 30 | 4 | -2497.056865 | 30 | 10 | 13706.29 |
