# Supplementary material for: Understanding the cryptic introgression and mixed ancestry of Red Junglefowl in India
Source: PLoS One. 2018 Oct 11;13(10):e0204351. doi: 10.1371/journal.pone.0204351 (PMC6188471; doi:10.1371/journal.pone.0204351)
Supplement: S5 Table — (DOC) [file pone.0204351.s005.doc]

**Table S5 The individual's proportion of membership^#^ of the wild RJFs (n=55) in each of the four clusters (*K* =4) inferred by Geneland analysis using uncorrelated allele frequency model**.

^#^ The proportion of membership of the best run showing the highest average logarithm of the posterior probability (see table S2- run 28 in uncorrelated allele frequency model that generated the highest average logarithm 2286.386865 at K 4).

| Sample ID | Sampling location | | Inferred cluster | | | |
| --- | --- | --- | --- | --- | --- | --- |
|  | Longitude | Latitude | I | II | III | IV |
| RJ-85_J&K_North | 497859.2437 | 3619192.996 | 0.185 | 0.172 | 0.181 | **0.463** |
| RJ-90_J&K_North | 512877.8384 | 3619478.35 | 0.185 | 0.172 | 0.181 | **0.463** |
| RJ-78_HP_North | 739667.4022 | 3369283.762 | 0.185 | 0.172 | 0.181 | **0.463** |
| RJ-79_HP_North | 739642.0565 | 3369221.647 | 0.185 | 0.172 | 0.181 | **0.463** |
| RJ-67_UK_North | 787953.5721 | 3333473.113 | 0.185 | 0.172 | 0.181 | **0.463** |
| RJ-68_UK_North | 800251.8779 | 3317804.947 | 0.185 | 0.172 | 0.181 | **0.463** |
| RJ-74_UK_North | 878191.6725 | 3264145.7 | 0.185 | 0.172 | 0.181 | **0.463** |
| RJ-75_UK_North | 785859.8337 | 3354129.227 | 0.185 | 0.172 | 0.181 | **0.463** |
| RJ-76_UK_North | 785858.2219 | 3354190.763 | 0.185 | 0.172 | 0.181 | **0.463** |
| RJ-77_UK_North | 786046.0801 | 3354164.951 | 0.185 | 0.172 | 0.181 | **0.463** |
| RJ-80_UK_North | 786127.8484 | 3354105.405 | 0.185 | 0.172 | 0.181 | **0.463** |
| RJ-84_UK_North | 919113.59 | 3240500.962 | 0.185 | 0.172 | 0.181 | **0.463** |
| RJ-116_UP_North | 1026200.973 | 3143166.564 | **0.485** | 0.194 | 0.154 | 0.167 |
| RJ-120_UP_North | 1023270.838 | 3140162.33 | **0.485** | 0.194 | 0.154 | 0.167 |
| RJ-123_UP_North | 1112663.955 | 3141360.868 | **0.485** | 0.194 | 0.154 | 0.167 |
| RJ-172_UP_North | 1031066.999 | 3133462.936 | **0.485** | 0.194 | 0.154 | 0.167 |
| RJ-51_UP_North | 985459.294 | 3165822.402 | **0.487** | 0.194 | 0.152 | 0.167 |
| RJ-52_UP_North | 986661.2122 | 3165779.659 | **0.485** | 0.194 | 0.154 | 0.167 |
| RJ-53_UP_North | 987365.9165 | 3167198.895 | **0.485** | 0.194 | 0.154 | 0.167 |
| RJ-54_UP_North | 988219.4165 | 3168315.511 | **0.485** | 0.194 | 0.154 | 0.167 |
| RJ-55_UP_North | 983430.2765 | 3171359.713 | **0.485** | 0.194 | 0.154 | 0.167 |
| RJ-56_UP_North | 984154.0488 | 3170339.483 | **0.485** | 0.194 | 0.154 | 0.167 |
| RJ-57_UP_North | 984467.5452 | 3170012.782 | **0.485** | 0.194 | 0.154 | 0.167 |
| RJ-58_UP_North | 984421.5806 | 3169146.04 | **0.485** | 0.194 | 0.154 | 0.167 |
| RJ-59_UP_North | 984265.0394 | 3169633.686 | **0.485** | 0.194 | 0.154 | 0.167 |
| RJ-60_UP_North | 984555.146 | 3169213.282 | **0.485** | 0.194 | 0.154 | 0.167 |
| RJ-61_UP_North | 984784.394 | 3168944.805 | **0.485** | 0.194 | 0.154 | 0.167 |
| RJ-62_UP_North | 983450.8702 | 3182695.739 | **0.485** | 0.194 | 0.154 | 0.167 |
| RJ-63_UP_North | 983322.2679 | 3181856.464 | **0.485** | 0.194 | 0.154 | 0.167 |
| RJ-64_UP_North | 983255.7421 | 3180834.416 | **0.485** | 0.194 | 0.154 | 0.167 |
| RJ-65_UP_North | 984462.7808 | 3181286.193 | **0.485** | 0.194 | 0.154 | 0.167 |
| RJ-66_UP_North | 987196.4585 | 3182357.772 | **0.485** | 0.194 | 0.154 | 0.167 |
| RJ-115_BH_East | 1527968.809 | 2741466.004 | 0.185 | 0.172 | 0.181 | **0.463** |
| RJ-98_BH_East | 1385052.198 | 3065144.488 | 0.185 | 0.172 | 0.181 | **0.463** |
| RJ-99_BH_East | 1385129.186 | 3063312.614 | 0.185 | 0.172 | 0.181 | **0.463** |
| RJ-101_BH_East | 1366229.277 | 2783600.145 | 0.185 | 0.172 | 0.181 | **0.463** |
| RJ-104_BH_East | 1660910.764 | 2817033.947 | 0.185 | 0.172 | 0.181 | **0.463** |
| RJ-105_BH_East | 1661486.536 | 2816989.222 | 0.185 | 0.172 | 0.181 | **0.463** |
| RJ-108_BH_East | 1386875.944 | 3066055.422 | 0.185 | 0.172 | 0.181 | **0.463** |
| RJ-109_BH_East | 1660226.173 | 2816347.547 | 0.185 | 0.172 | 0.181 | **0.463** |
| RJ-110_SK_East | 2243319.789 | 3110198.667 | 0.154 | 0.214 | **0.463** | 0.170 |
| RJ-3_CH_Cent-SE | 1265367.71 | 2257731.862 | 0.185 | 0.172 | 0.181 | **0.463** |
| RJ-5_CH_Cent-SE | 1273685.02 | 2242450.812 | 0.185 | 0.172 | 0.181 | **0.463** |
| RJ-7_OR_Cent-SE | 2194188.106 | 2978374.053 | 0.185 | 0.172 | 0.181 | **0.463** |
| RJ-8_OR_Cent-SE | 1419746.887 | 2216713.527 | 0.185 | 0.172 | 0.181 | **0.463** |
| RJ-10_OR_Cent-SE | 1742074.552 | 2336935.039 | 0.185 | 0.172 | 0.181 | **0.463** |
| RJ-11_AS_NorthEast | 2102878.023 | 3054533.549 | 0.185 | 0.172 | 0.181 | **0.463** |
| RJ-113_AS_NorthEast | 2295200.951 | 2847341.518 | 0.165 | 0.227 | **0.432** | 0.176 |
| RJ-173_MN_NorthEast | 2450071.728 | 2943463.726 | 0.174 | **0.425** | 0.196 | 0.205 |
| RJ-174_MN_NorthEast | 2419524.448 | 2858305.315 | 0.174 | **0.425** | 0.196 | 0.205 |
| RJ-175_MN_NorthEast | 2419423.532 | 2858388.449 | 0.174 | **0.425** | 0.196 | 0.205 |
| RJ-176_MN_NorthEast | 2418723.168 | 2857672.144 | 0.174 | **0.425** | 0.196 | 0.205 |
| RJ-6_MG_NorthEast | 2037685.192 | 2939281.977 | 0.185 | 0.172 | 0.181 | **0.463** |
| RJ-112_MZ_NorthEast | 2353317.156 | 2673811.54 | 0.185 | 0.172 | 0.183 | **0.460** |
| RJ-114_NG_NorthEast | 2378830.557 | 2941320.87 | 0.163 | 0.209 | **0.419** | 0.209 |
