# Supplementary figures and images for: Understanding the cryptic introgression and mixed ancestry of Red Junglefowl in India
Source: PLoS One. 2018 Oct 11;13(10):e0204351. doi: 10.1371/journal.pone.0204351 (PMC6188471; doi:10.1371/journal.pone.0204351)

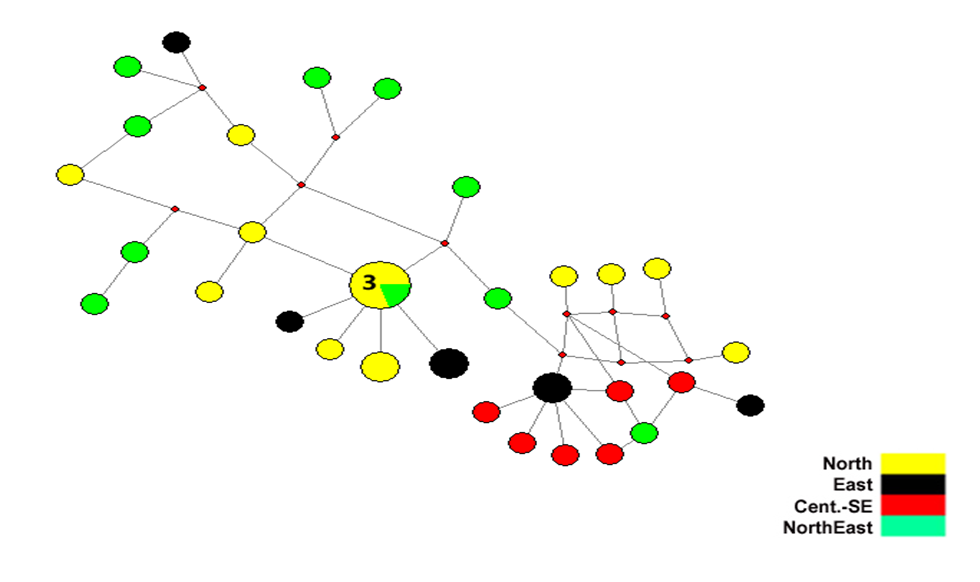

Supplement: S1 Fig — (Haplotypes of four RJF populations are represented in different colored circles. The size of the circle is proportional to the haplotype frequency. Haplotype 3 is shared between North and Northeast populations). (TIF) [file pone.0204351.s006.tif]

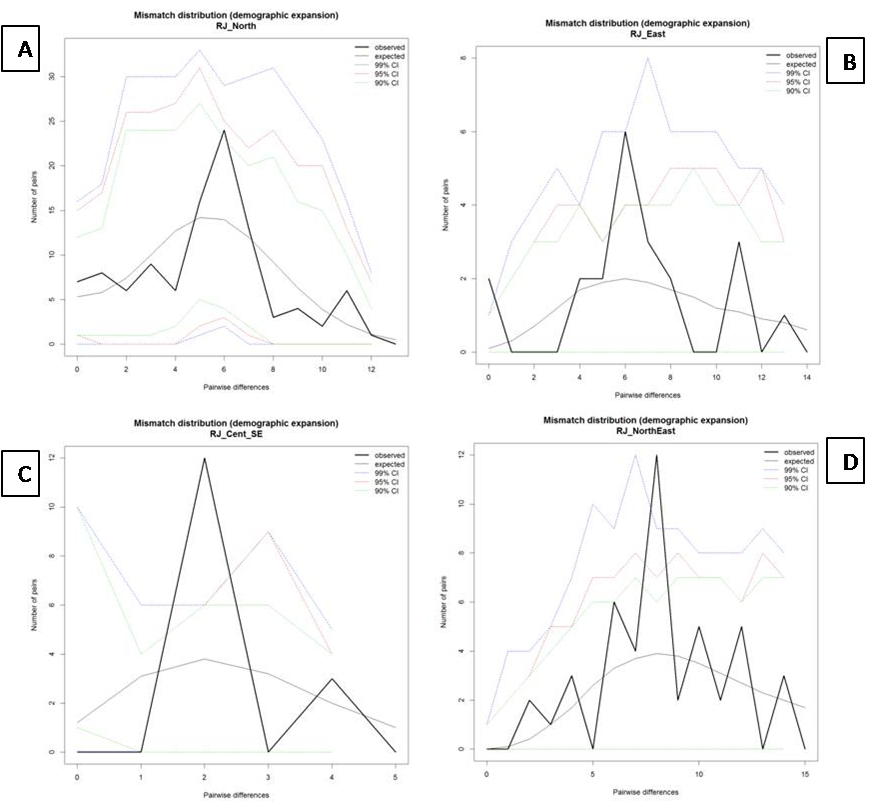

Supplement: S2 Fig — A, North; B, East; C, Central-Southeast and D,Northeast. (TIF) [file pone.0204351.s007.tif]

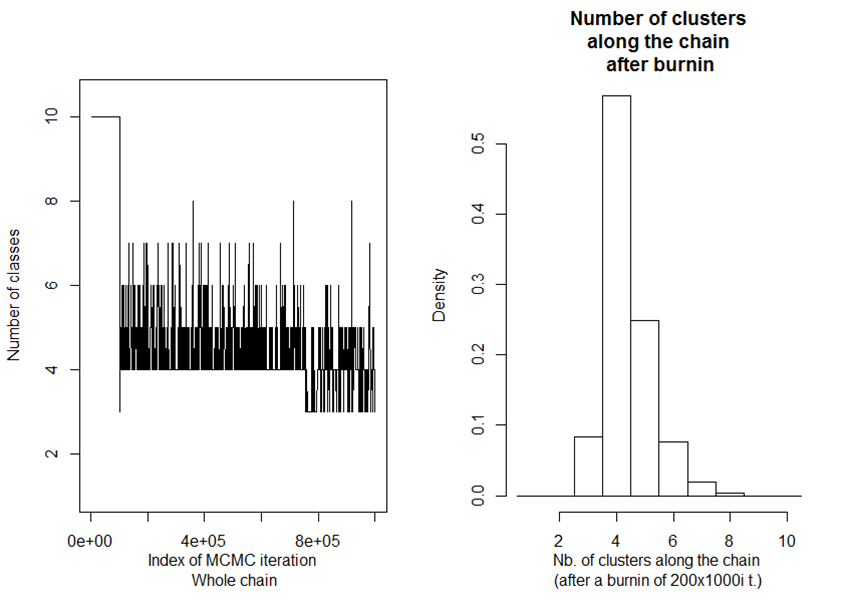

Supplement: S3 Fig — Analysis was conducted under the spatial model with uncorrelated allele frequencyincorporating latitude/longitude. (TIF) [file pone.0204351.s008.tif]
